# Interplay between soluble CD74 and macrophage-migration inhibitory factor drives tumor growth and influences patient survival in melanoma

**DOI:** 10.1038/s41419-022-04552-y

**Published:** 2022-02-04

**Authors:** Yasunari Fukuda, Matias A. Bustos, Sung-Nam Cho, Jason Roszik, Suyeon Ryu, Victor M. Lopez, Jared K. Burks, Jeffrey E. Lee, Elizabeth A. Grimm, Dave S. B. Hoon, Suhendan Ekmekcioglu

**Affiliations:** 1 Department of Melanoma Medical Oncology, The University of Texas MD Anderson Cancer Center, Houston, TX 77030 USA; 2 Department of Translational Molecular Medicine, Saint John’s Cancer Institute, Providence Saint John’s Health Center, Santa Monica, CA 90404 USA; 3 Department of Genomic Medicine, The University of Texas MD Anderson Cancer Center, Houston, TX 77030 USA; 4 Department of Genome Sequencing, Saint John’s Cancer Institute, Providence Saint John’s Health Center, Santa Monica, CA 90404 USA; 5 Department of Leukemia, The University of Texas MD Anderson Cancer Center, Houston, TX 77030 USA; 6 Department of Surgical Oncology, The University of Texas MD Anderson Cancer Center, Houston, TX 77030 USA

**Keywords:** Melanoma, Prognostic markers

## Abstract

Soluble forms of receptors play distinctive roles in modulating signal-transduction pathways. Soluble CD74 (sCD74) has been identified in sera of inflammatory diseases and implicated in their pathophysiology; however, few relevant data are available in the context of cancer. Here we assessed the composition and production mechanisms, as well as the clinical significance and biological properties, of sCD74 in melanoma. Serum sCD74 levels were significantly elevated in advanced melanoma patients compared with normal healthy donors, and the high ratio of sCD74 to macrophage-migration inhibitory factor (MIF) conferred significant predictive value for prolonged survival in these patients (*p* = 0.0035). Secretion of sCD74 was observed primarily in melanoma cell lines as well as a THP-1 line of macrophages from monocytes and primary macrophages, especially in response to interferon-γ (IFN-γ). A predominant form that showed clinical relevance was the 25-KDa sCD74, which originated from the 33-KDa isoform of CD74. The release of this sCD74 was regulated by either a disintegrin and metalloproteinase-mediated cell-surface cleavage or cysteine-protease-mediated lysosomal cleavage, depending on cell types. Both recombinant and THP-1 macrophage-released endogenous sCD74 suppressed melanoma cell growth and induced apoptosis under IFN-γ stimulatory conditions via inhibiting the MIF/CD74/AKT-survival pathway. Our findings demonstrate that the interplay between sCD74 and MIF regulates tumor progression and determines patient outcomes in advanced melanoma.

## Introduction

Inflammation is a hallmark of various types of cancers, including melanoma [1]. Inflammation is involved in multistage carcinogenesis, tumorigenesis, and tumor resistance. A plethora of intrinsic molecules and structural components released by either tumor cells or surrounding cells evokes a wide range of inflammatory responses. These inflammatory stimuli successively alter the phenotypic and functional characteristics of cancer cells to be more vulnerable toward tumor progression [2, 3].

Our group has a significant experience investigating the impact of inflammatory pathways on the regulation of the tumor and tumor immune microenvironment (TIME) in melanoma [4–7]. CD74 is a type-II transmembrane glycoprotein that has been shown to have two biological functions. First, CD74 functions as a chaperone and a transport cofactor for major histocompatibility complex class II, responsible for appropriate antigen presentation [8]. Second, a small fraction of free CD74 displayed on the cell surface serves as a receptor for macrophage-migration inhibitory factor (MIF) [9]. The MIF–CD74 axis has been shown to play pivotal roles in not only initiating an oncogenic signaling pathway, but also provoking inflammatory responses, thereby promoting tumor growth and an immunosuppressive milieu [10–13]. Previous work by our group showed that autocrine MIF–CD74 interactions in response to interferon-γ (IFN-γ) enhanced the phospho-AKT (pAKT), which sequentially induced the expression of inflammatory cytokines and tumor progression in melanoma [5]. Conversely, we have recently demonstrated that high expression of CD74 in the tumors showed the most promise, among a set of inflammatory protein markers tested, as a favorable survival predictor in advanced melanoma [6, 7]. Our findings point to CD74 as an intriguing molecule possessing multifaceted functions in melanoma, some contradictory, depending on the context.

The soluble form of receptors can be generated mainly by two mechanisms, namely proteolytic cleavage and alternative RNA splicing [14–18]. Mounting evidence has shown that the soluble receptor molecules exhibit specific biological activities distinct from those of the membrane-anchored form, and these activities may be associated with a wide spectrum of diseases [14, 19]. Although soluble CD74 (sCD74) has already been quantified in human plasma more than two decades ago [20], the biological function and clinical relevance of sCD74 have not been intensively investigated until recently. Assis et al. first demonstrated that circulating sCD74 and MIF profiles helped distinguish primary biliary cirrhosis from the more inflammatory phenotype of acute immune hepatitis [21]. Wu et al. revealed that sCD74 was released by alveolar macrophages under MIF stimulatory conditions and could induce the production of inflammatory cytokines in a mouse model of lipopolysaccharide-induced acute lung injury [22]. Overall, sCD74 may participate in the regulation of tumor progression and an inflammatory milieu; however, to the best of our knowledge, no relevant data on sCD74 in the context of cancer are available so far. In this study, we sought to determine the clinical implications of sCD74 and their biological functions upon interaction with MIF in melanoma.

## Materials and methods

### Cell lines and cell culture

BRAF-mutant melanoma cell line A375 (CVCL_0132), NRAS-mutant melanoma line SK-MEL-2 (CVCL_0069), BRAF/NRAS wild-type melanoma line MeWo (CVCL_0445), and monocyte-like cell line THP-1 (CVCL_0006) were purchased from the American Type Culture Collection (Manassas, VA, USA). NRAS-mutant melanoma line SB2 (CVCL_0516) was generously provided by Dr. Michael Davies at The University of Texas MD Anderson Cancer Center (Houston, TX, USA). Peripheral blood primary CD4^+^ T cells, CD8^+^ T cells, B cells, natural killer (NK) cells, and M0 macrophages (MΦ) were purchased from Stemcell Technologies (Vancouver, British Columbia, Canada). All cells were cultured in Roswell Park Memorial Institute 1640 supplemented with 10% heat-inactivated fetal bovine serum (FBS), 100 U/mL penicillin, and 100 µg/mL streptomycin. To induce differentiation into M0 MΦ from THP-1, we cultured THP-1 in medium with 200 ng/mL phorbol 12-myristate 13-acetate (PMA) for 48 h, and then sequentially replaced the medium with fresh medium without PMA and cultured the cells for another 24 h. For primary M0 MΦ, we cultivated them for 7 days until all cells were attached on the plate and then used for further assays. Cell cultures were maintained in 5% CO_2_ at 37 °C. All the cell lines had been authenticated using short tandem-repeat DNA fingerprinting using the AmpFLSTR Identifiler kit, within the last 3 years. All experiments were performed with mycoplasma-free cell lines.

### Patient clinical samples

Serum and plasma samples were collected at the initial diagnosis from two independent cutaneous melanoma patient cohorts [5, 6]. Cohort 1 [5] included melanoma patients at stage 0, I, II, or III according to the 7^th^ edition of AJCC staging system (*n* = 47), and cohort 2 [6] included only stage-IIIB/C advanced melanoma patients (*n* = 23). Serum samples from age-matched normal healthy donors (NHDs, *n* = 34) were also employed. Clinicopathological characteristics for the melanoma patients and demographic information for the NHDs are summarized in Supplementary Table S1. Immunohistochemically (IHC) stained slides from two cohorts were used to assess the association between CD74 and MIF expressions in tumor tissues and sCD74 and MIF levels in paired sera, respectively. The definition of low and high CD74 and MIF expressions in tumor tissues was based on our previous reports [5, 6]. Briefly, for each patient sample, two independent readings were performed, and any discrepancies in scores were subsequently reconciled. Intensity-scoring staining was defined as follows: “0,” none; “1,” light; “2,” moderate; and “3,” intense. Then, we categorized “0” or “1” as low expression and “2” or “3” as high expression. In cohort 2, one patient was lost on follow-up, leaving 22 available patients for survival analysis. The use of clinical samples was approved by the Institutional Review Board of MD Anderson Cancer Center (no. LAB01-717). Informed consent forms were signed by each patient before participation.

Additional materials and methods are provided in Supplementary Appendix.

### Statistical analysis

Statistical analyses were conducted using GraphPad Prism 8 (GraphPad Software Inc., San Diego, CA, USA) or JMP software (SAS Inc., Cary, NC, USA). Continuous variables were expressed as the median (95% confidence interval) or mean ± standard deviation (SD), as appropriate. Variables were compared between groups using the Student *t*-test or Wilcoxon signed-rank test as appropriate. The Pearson correlation coefficient was used to measure the strength of a linear association between two variables. Receiver-operating characteristic (ROC) analyses were used to determine the optimal cutoff values for sCD74, MIF, and sCD74/MIF ratio for the evaluation of survival. Survival curves were estimated using the Kaplan–Meier method and compared using the log-rank test. *P-*values of <0.05 were considered statistically significant. All in vitro experiments were carried out at least in duplicate, and representative results are shown in the figures.

## Results

### High serum sCD74 to MIF ratio was associated with favorable patient survival

**Fig. 1:**
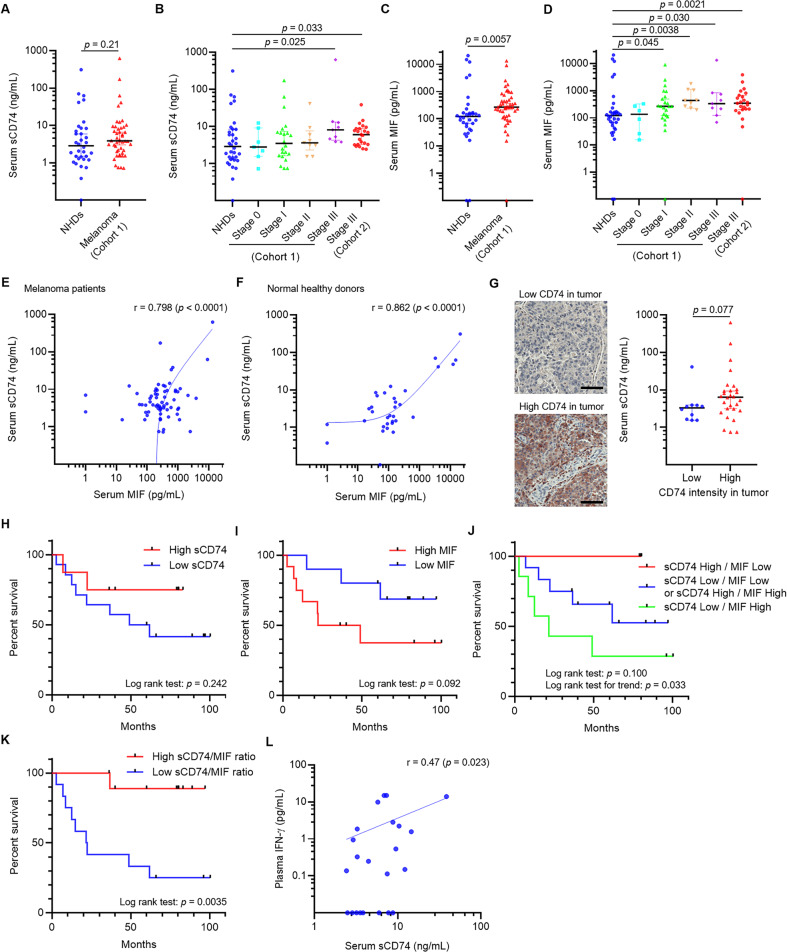
Serological sCD74 and MIF levels and their association with melanoma patient survival. **A** Serum sCD74 levels in all melanoma patients in cohort 1 and NHDs. **B** Serum sCD74 levels in melanoma patients in cohort 1 by stage, cohort 2, and NHDs. **C** Serum MIF levels in all melanoma patients in cohort 1 and NHDs. **D** Serum MIF levels in melanoma patients in cohort 1 by stage, cohort 2, and NHDs. **E** Correlation between serum sCD74 and MIF levels in melanoma patients (cohort 1 + cohort 2). **F** Correlation between serum sCD74 and MIF levels in NHDs. **G** Representative images of low and high CD74 intensity (left) in tumor. Box plot of serum sCD74 levels (right) in patients with low and high CD74 intensity in tumor in cohort 1. Scale bar = 100 μm. **H**–**K** Kaplan–Meier curves show post-blood-test OS in cohort-2 patients with stage-IIIB/C melanoma based on serum sCD74 levels (**H**), where low sCD74: <7.76 ng/mL (*n* = 15), high sCD74: ≥7.76 ng/mL (*n* = 7); serum MIF levels (**I**), where low MIF: < 264.59 pg/mL (*n* = 10), high MIF: ≥ 264.59 pg/mL (*n* = 12); combination of serum sCD74 and MIF levels (**J**), where high sCD74 (≥7.76 ng/mL) and low MIF ( < 264.59 pg/mL) (*n* = 3, red line), low sCD74 (<7.76 ng/mL) and low MIF (<264.59 pg/mL) or high sCD74 (≥7.76 ng/mL) and high MIF (≥264.59 pg/mL) (*n* = 12, blue line), or low sCD74 (<7.76 ng/mL) and high MIF (≥264.59 pg/mL) (*n* = 7, green line); and ratio of sCD74 (pg/mL) to MIF (pg/mL) (**K**), where low sCD74/MIF: <26.97 (*n* = 12), high sCD74/MIF: ≥26.97 (*n* = 10). Patients were grouped according to the optimal cutoff values determined by ROC analyses, and significance of survival was determined by log-rank test. **L** Correlation between serum sCD74 levels and plasma IFN-γ levels in cohort 2 melanoma patients. Significance in difference between two groups was tested by Wilcoxon signed-rank test. The Pearson correlation coefficient was used to measure the strength of a linear association between serum sCD74 and MIF levels, as well as serum sCD74 levels and plasma IFN-γ levels. IFN-γ interferon-γ, MIF macrophage-migration inhibitory factor, NHD normal healthy donor, ROC receiver-operating curve, OS overall survival.

Serum concentrations of sCD74 and MIF in two independent cohorts of melanoma patients [5, 6] and in NHDs were measured. Whereas serum sCD74 levels were similar between melanoma patients at all stages and NHDs (Fig. 1A), the levels of sCD74 were significantly higher in stage-III patients in cohort 1 compared with NHDs (Fig. 1B). On the other hand, serum MIF levels were enriched in melanoma patients in cohort 1 when considered separately by stage, as well as in all melanoma patients compared with NHDs (Fig. 1C, D). We validated that serum sCD74 and MIF levels were elevated in patients with stage-IIIB/C melanoma in cohort 2 compared with NHDs (Fig. 1B, D). sCD74 and MIF levels were positively correlated in both melanoma patients and NHDs (Fig. 1E, F). We also observed higher sCD74 levels in patients whose melanoma tissues had high CD74 staining intensity compared with those whose melanoma had low CD74 expression, although the difference was not statistically significant (Fig. 1G, *p* = 0.077). On the contrary, MIF staining intensity in tumor tissues was not associated with paired-serum MIF levels (Supplementary Fig. 1).

Next, we analyzed the associations between serum sCD74 and MIF levels and overall survival (OS) in stage-IIIB/C melanoma patients in cohort 2. OS was calculated from the day of blood draw to the day of patient death or last visit. When patients were divided into two groups according to the optimal cutoff values of sCD74 and MIF determined by ROC analyses (7.76 ng/mL for sCD74, and 264.59 pg/mL for MIF), patients with high sCD74 and low MIF levels had better OS compared with those with low sCD74 and high MIF levels, respectively, although these differences did not reach statistical significance (Fig. 1H, I). In addition, three categories were generated according to the above-mentioned cutoff values, one for patients with high sCD74 and low MIF levels, a second for patients with low sCD74 and low MIF levels or high sCD74 and high MIF levels, and a third for patients with low sCD74 and high MIF levels (Fig. 1J). Although survival differences across three groups also did not reach statistical significance (*p* = 0.100), log-rank test for trend was significant (*p* = 0.033). However, when patients were classified into two groups by the optimal value of the sCD74 to MIF ratio determined by ROC analyses (sCD74 level [pg/mL] divided by MIF level [pg/mL]), a high sCD74/MIF ratio (≥26.97) was associated with significantly improved OS compared with that of a low sCD74/MIF ratio (<26.97) (Fig. 1K, *p* = 0.0035). Altogether, these results suggest the possible interaction between sCD74 and MIF, which may influence tumor progression in melanoma.

### Soluble form of CD74 was released from melanoma cells and macrophages

We sought the possible cellular sources of sCD74 in melanoma based on the theoretical assumption that sCD74 is released from CD74-expressing cells. We chose melanoma cells and immune cells as potential candidates since CD74 is expressed in both tumor tissue (Fig. 1G) and tumor microenvironment [6]. In addition, The Cancer Genome Atlas (TCGA) and The Genotype-Tissue Expression (GTEx) RNA-sequencing data showed that CD74 mRNA expression in spleen and melanoma tissues was higher than that in normal tissues from other organs (Supplementary Fig. 2A).

**Fig. 2:**
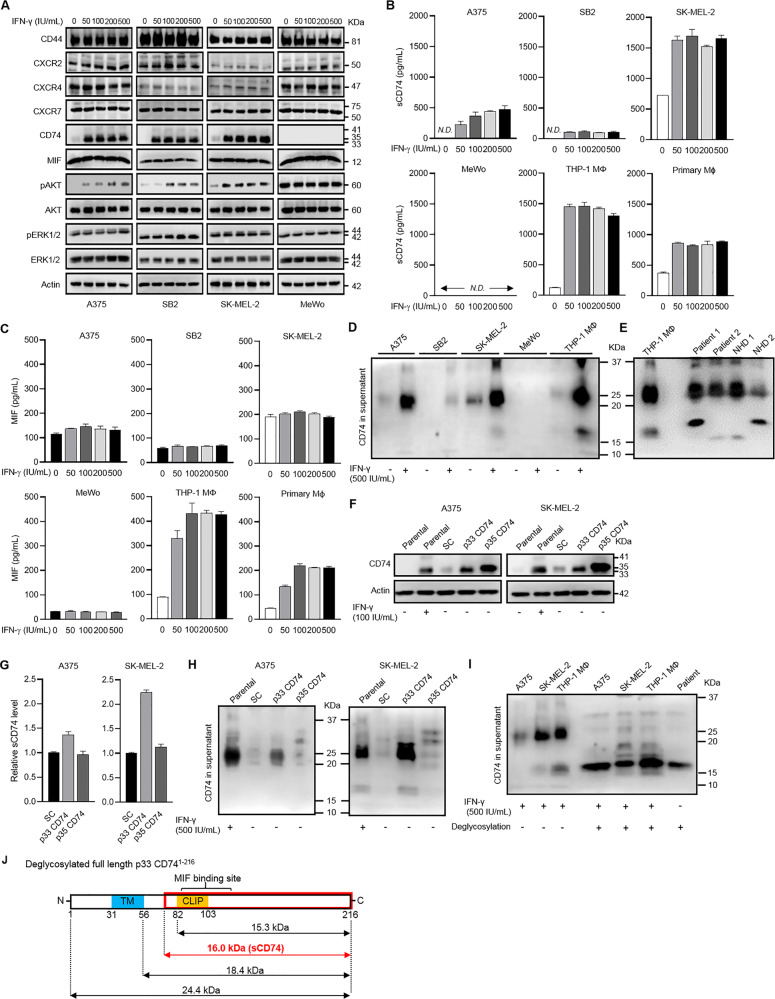
CD74 expression and release of sCD74 from melanoma cell lines and immune cells. **A** WB analysis of changes in CD44, CXCR2, CXCR4, CXCR7, CD74, MIF, pAKT, AKT, pERK1/2, and ERK1/2 expressions in response to IFN-γ (0–500 IU/mL) in A375, SB2, SK-MEL-2, and MeWo. Actin, AKT, and ERK1/2 were used as loading controls. **B** Release of sCD74 in supernatants 24 h after IFN-γ stimulation (0–500 IU/mL) in A375, SB2, SK-MEL-2, MeWo, THP-1 MΦ, and primary MΦ measured by ELISA (*n* = 3). **C** Release of MIF in supernatants 24 h after IFN-γ stimulation (0–500 IU/mL) in A375, SB2, SK-MEL-2, MeWo, THP-1 MΦ, and primary MΦ measured by ELISA (*n* = 4). **D** WB analysis of sCD74 in supernatants of A375, SB2, SK-MEL-2, MeWo, and THP-1 MΦ with or without 500 IU/mL IFN-γ stimulation. **E** WB analysis of sCD74 in the sera of 2 melanoma patients and 2 NHDs. Supernatant of THP-1 MΦ after 500 IU/mL IFN-γ stimulation was used as a reference control. **F** WB analysis of CD74 in A375 and SK-MEL-2 infected with lentivirus-expressing SC, p33 CD74, and p35 CD74. Parental cells with or without 100 IU/mL IFN-γ stimulation were used as controls. **G** Release of sCD74 in supernatants under basal conditions in A375 and SK-MEL-2 infected with lentivirus-expressing SC, p33 CD74, and p35 CD74 measured by ELISA (*n* = 3), and the fold-change relative to sCD74 levels in supernatants of SC cells is shown as bar graphs. **H** WB analysis of sCD74 in supernatants of A375 and SK-MEL-2 infected with lentivirus-expressing SC, p33, and p35 CD74. Parental cells under 500 IU/mL IFN-γ stimulation were used as a control. **I** WB analysis of sCD74 in supernatants of A375, SK-MEL-2, and THP-1 MΦ with or without deglycosylation treatment under 500 IU/mL IFN-γ stimulatory conditions. Serum of a melanoma patient was also deglycosylated. **J** Schematic illustration of deglycosylated full-length p33 CD74^1-216^. Considering that MW of deglycosylated sCD74 was approximately 16 KDa, sCD74 was equivalent to a part of full-length CD74 (red box). Graph values represent mean ± SD. CLIP class-II-associated invariant chain peptide, ELISA enzyme-linked immunosorbent assay, IFN-γ interferon-γ, MW molecular weight, MΦ macrophage, NHD normal healthy donor*,* N.D. not detectable, SC scramble, SD standard deviation, TM transmembrane, WB Western blot.

First, we investigated CD74 expression in four melanoma cell lines (A375, SB2, SK-MEL-2, and MeWo) as well as five major immune-cell types (THP-1 MΦ cell line and primary culture CD4^+^ T cells, CD8^+^ T cells, B cells, NK cells, and MΦ) with or without IFN-γ stimulation, as IFN-γ is a strong inducer of CD74 expression [5]. Only primary B cells and NK cells constitutively expressed CD74 (Supplementary Fig. 2B). A375, SB2, SK-MEL-2, THP-1 MΦ, and primary MΦ scarcely expressed total and cell-surface CD74 under basal conditions; however, IFN-γ stimulation remarkably upregulated these expression levels (Fig. 2A, Supplementary Fig. 2B, C). On the other hand, MeWo and primary CD4^+^ T cells, CD8^+^ T cells were completely negative for CD74, even after IFN-γ stimulation (Fig. 2A and Supplementary Fig. 2B). Subsequently, we quantified the release of sCD74 by enzyme-linked immunosorbent assay (ELISA). sCD74 was detectable in supernatants of SK-MEL-2 as well as THP-1 MΦ and primary MΦ under basal conditions and dramatically increased in response to IFN-γ in A375, SB2, SK-MEL-2, THP-1 MΦ, and primary MΦ (Fig. 2B). Consistently, serum sCD74 levels in melanoma patients (cohort 2) were positively associated with plasma IFN-γ levels (Fig. 1L). In contrast, release of sCD74 from primary NK cells was low, despite IFN-γ stimulation, and stimulation with any concentration of IFN-γ could not induce sCD74 release in MeWo, primary CD4^+^ T cells, and CD8^+^ T cells (Fig. 2B and Supplementary Fig. 2D). Surprisingly, sCD74 was also undetectable in the supernatant of primary B cells, despite their constitutive CD74 expression (Supplementary Fig. 2D). Combined stimulation with IL-4 and IL-13 did not induce CD74 expression and sCD74 release in THP-1 MΦ and primary MΦ (Supplementary Fig. 2E, F), indicating that sCD74 was much likely produced by M1-like MΦ than by M2-like MΦ. We also evaluated the release of MIF in supernatants of A375, SB2, SK-MEL-2, MeWo, THP-1 MΦ, and primary MΦ (Fig. 2C) and found that IFN-γ stimulation upregulated the release only in THP-1 MΦ and primary MΦ. Interestingly, it seems likely that the sCD74 levels released in supernatants were positively related to that of MIF in these cells (Fig. 2B, C), which is consistent with our clinical results (Fig. 1E, F).

Next, we assessed the components of sCD74 by western blotting (WB) using an anti-CD74 antibody (Ab) against the ectodomain part of CD74. An approximately 25-KDa band was distinctly detectable in supernatants of A375, SK-MEL-2, and THP-1 MΦ, with an additional band of 16 KDa in supernatants of SK-MEL-2 and THP-1 MΦ under IFN-γ-stimulated conditions (Fig. 2D). The 25-kDa band was also found in sera of both melanoma patients and NHDs (Fig. 2E); furthermore, some sera included two different bands (18- and 15 KDa) that were undetectable in supernatants of any cell lines used in this study (Fig. 2D, E and Supplementary Fig. 2G). Importantly, the intensity of the 25-KDa bands exhibited linear correlation with serum sCD74 levels measured by ELISA (Supplementary Fig. 2G). Conversely, no clear bands were detected from cell culture supernatants and serum samples when an anti-CD74 Ab against the cytoplasmic tail of CD74 was used (data not shown).

To identify CD74 isoforms responsible for the generation of sCD74, we established A375 and SK-MEL-2 melanoma cells stably overexpressing the p33 and p35 isoforms of CD74, which are the main isoforms of CD74 in these cells (Fig. 2A). WB analysis confirmed that p33 CD74 and p35 CD74 were overexpressed in both cell lines under basal conditions (Fig. 2F); however, cell-surface CD74-positive cells were abundantly found in p33 CD74-overexpressing cells (Supplementary Fig. 2H), possibly because of the presence of endoplasmic reticulum retention motif in p35 isoform [23]. In supernatants, the release of 25-KDa sCD74 was predominantly elevated in p33 CD74-overexpressing cells (Fig. 2G, H), suggesting that the p33 isoform of CD74 is a predominant origin of sCD74.

Furthermore, to determine whether sCD74 was glycosylated, we exposed supernatants of A375, SK-MEL-2, THP-1 MΦ, and a patient’s serum to deglycosylases to induce deglycosylation. After treatment with deglycosylases, the sCD74 showed a large drop in molecular weight (MW) from 25 KDa to 16 KDa in all cell lines and in a patient’s serum (Fig. 2I). Given that there is 8.6-KDa difference in size between glycosylated and deglycosylated full-length p33 CD74 (MWs of 33- and 24.4 KDa, respectively), the 16-KDa sCD74 was identical to the deglycosylated product of the glycosylated 25-KDa sCD74. We found that this 16-KDa sCD74 corresponded to a part of CD74, ranging from somewhere between the transmembrane and class-II-associated invariant chain peptide (CLIP) region to the C terminus and containing the MIF-binding site (Fig. 2J). We cannot rule out the possibility that this 25-KDa band is attributed to deglycosylated full-length p33 CD74 whose MW is 24.4 KDa (Fig. 1J); however, when cell culture supernatants were treated with deglycosylases, approximately 25-KDa bands almost disappeared (Fig. 2I), suggesting that deglycosylated full-length p33 CD74 is not a major component of sCD74, even if it exists in supernatants.

### Release of sCD74 was regulated by ADAM-mediated cell-surface cleavage or cysteine-protease-mediated lysosomal cleavage

**Fig. 3:**
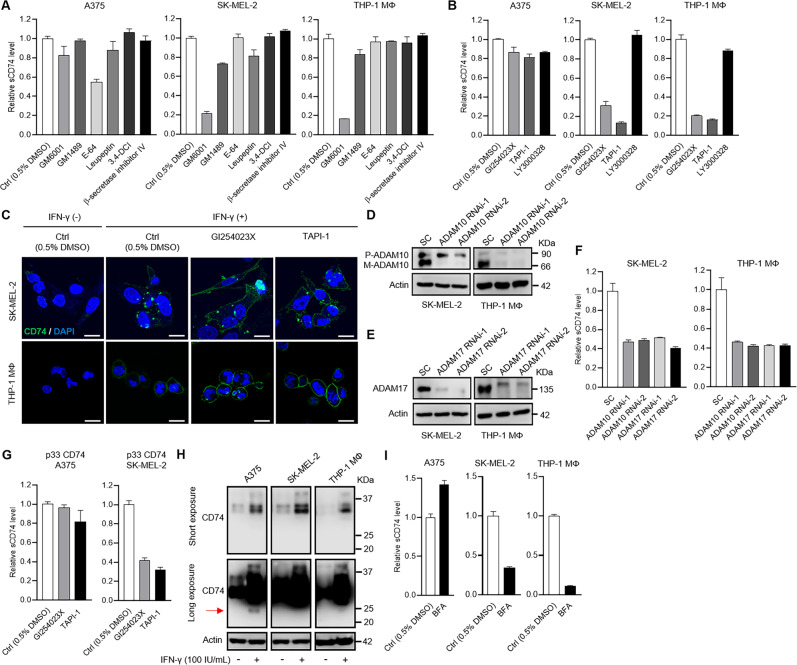
Protease-inhibitory assay in melanoma cells and THP-1-derived macrophages. **A**, **B** A375, SK-MEL-2, and THP-1 MΦ were treated with broad protease inhibitors (**A**), including GM6001 (MMP and ADAM inhibitor), GM1489 (MMP inhibitor), E-64 (cysteine inhibitor), leupeptin (serine, cysteine, and threonine inhibitor), 3,4-DCI (serine inhibitor), and β-secretase inhibitor IV (BACE inhibitor) or selective inhibitors (**B**), including GI254023X (ADAM10 inhibitor), TAPI-1 (ADAM17 inhibitor), and LY3000328 (cathepsin-S inhibitor) under 500 IU/mL IFN-γ stimulatory conditions. Release of sCD74 in supernatants was measured by ELISA (*n* = 3), and the fold change relative to sCD74 levels in supernatants of cells treated with 0.5% DMSO is shown as bar graphs. **C** Representative images of immunocytochemical staining of cell-surface CD74 in SK-MEL-2 and THP-1 MΦ treated with GI254023X and TAPI-1 under 500 IU/ml IFN-γ stimulation. Two cell lines were immunostained with CD74 (green) and DAPI (blue). Scale bar = 20 μm. **D**, **E** Efficacies of two individual siRNAs in knocking down ADAM10 expression (**D**) and ADAM17 expression (**E**) were analyzed by WB in SK-MEL-2 and THP-1 MΦ. SC siRNA was used as a control. **F** Release of sCD74 in supernatants was measured by ELISA in SK-MEL-2 and THP-1 MΦ transfected with SC siRNA, ADAM10 RNAi-1 and -2, and ADAM17 RNAi-1 and -2 under 500 IU/mL IFN-γ stimulatory conditions. Bar graphs show the fold change relative to sCD74 levels in supernatants of cells transfected with SC siRNA (*n* = 3). **G** A375 and SK-MEL-2 infected with lentivirus-expressing p33 CD74 were treated with GI254023X and TAPI-1 under basal conditions. Release of sCD74 in supernatants was measured by ELISA (*n* = 3), and the fold change relative to sCD74 levels in supernatants of cells treated with 0.5% DMSO is shown as bar graphs. **H** WB analysis of CD74 expression in A375, SK-MEL-2, and THP-1 MΦ with or without 100 IU/mL IFN-γ stimulation after a short exposure (upper) and a long exposure (lower). Cell lysates precipitated with acetone were subjected to WB analysis, and actin was used as a loading control. Arrow indicates 25-KDa bands. **I** A375, SK-MEL-2, and THP-1 MΦ were treated with 100 nM BFA for 24 h under 500 IU/mL IFN-γ-stimulated conditions. Release of sCD74 in supernatants was measured by ELISA (*n* = 3) and the fold change relative to sCD74 levels in supernatants of cells treated with 0.5% DMSO is shown as bar graphs. Graph values represent mean ± SD. ADAM a disintegrin and metalloproteinase, BFA brefeldin A, DAPI 4′,6-diamidino-2-phenylindole, DMSO dimethyl sulfoxide, ELISA enzyme-linked immunosorbent assay, IFN-γ interferon-γ, MMP matrix metalloproteinase, MΦ macrophage, SC scramble, SD standard deviation, siRNA short-interference RNA, WB Western blot, 3,4-DCI 3,4-dichloroisocoumarin.

The specific processes responsible for sCD74 production remain unknown. To assess the effect of proteolytic enzymes on CD74 shedding, we first treated sCD74-releasing A375, SK-Mel-2, and THP-1 MΦ cell lines with broad-spectrum protease inhibitors (Supplementary Table S2). In the presence of IFN-γ stimulation, E-64 (broad-spectrum cysteine inhibitor) suppressed sCD74 levels up to 50% in supernatants of A375 (Fig. 3A and Supplementary Fig. 3A), and GM6001 (broad-spectrum inhibitor of matrix metalloproteinase [MMP] and a disintegrin and metalloproteinase [ADAM]) reduced sCD74 levels up to 85% in supernatants of SK-MEL-2 and THP-1 MΦ (Fig. 3A).

We next focused on selective sheddases, including ADAM10, ADAM17, and cathepsin S. ADAM10 and ADAM17 are the most relevant enzymes involved in canonical shedding of substrates, and cathepsin S is known to be involved in truncating the N terminal of CLIP on lysosomal CD74. GI254023X (ADAM10 inhibitor), TAPI-1 (ADAM17 inhibitor), and LY3000328 (cathepsin-S inhibitor) slightly reduced sCD74 release from A375, while GI254023X and TAPI-1 dramatically reduced sCD74 release from SK-MEL-2 and THP-1 MΦ under IFN-γ-stimulated conditions (Fig. 3B and Supplementary Fig. 3B). Immunocytochemistry analysis showed that GI254023X and TAPI-1 increased cell-surface CD74 staining in SK-MEL-2 and THP-1 MΦ by inhibiting cell-surface cleavage (Fig. 3C). We also examined the influence of protease inhibitors on sCD74 secretion without IFN-γ stimulation in SK-MEL-2 and THP-1 MΦ, in which sCD74 was constitutively detectable (Fig. 2B). GM6001, GI254023X, and TAPI-1 suppressed sCD74 production to a similar extent to that seen in IFN-γ-stimulated conditions, indicating that IFN-γ did not alter susceptibility to the protease inhibitors (Supplementary Fig. 3C, D). Gene silencing by short-interfering RNA (siRNA) validated the effects of ADAM10 and ADAM17 on CD74 cleavage in these three cell lines (Fig. 3D–F, Supplementary Fig. 3E–G). In addition, GI254023X and TAPI-1 suppressed sCD74 secretion from SK-MEL-2, but not from A375, upon p33 CD74 overexpression under basal conditions (Fig. 3G).

Next, we examined the production mechanisms of sCD74 in A375. Considering that the release of sCD74 from this cell was suppressed by cysteine inhibitor E-64, we hypothesized that CD74 was cleaved mainly in the lysosome, as cysteine proteases are localized mainly in the lysosome. To address this, we first evaluated if the 25-KDa CD74 was present in cell lysates. Cell lysates precipitated with acetone were subjected to WB analysis. As shown in Fig. 3H, the 25-KDa band was detected only in cell lysate of A375 under IFN-γ-stimulated conditions. Next, cells were incubated with brefeldin A (BFA) under IFN-γ-stimulated conditions. sCD74 release in supernatants was blocked in SK-MEL-2 and THP-1 MΦ, but was unaffected in A375 (Fig. 3I and Supplementary Fig. 3H), most likely because BFA inhibited CD74 export from the Golgi compartment to the cell surface in SK-MEL-2 and THP-1 MΦ, but did not alter the function of lysosomes in A375. To exclude the possible existence of exosome-membrane-related CD74, which can be released in supernatants and captured by ELISA, we exposed the three cell lines to GW4869, an exosome-synthesis inhibitor, under IFN-γ-stimulated conditions. We found that GW4869 did not suppress sCD74 release in all cell lines (Supplementary Fig. 3I).

The soluble form of receptors also can be generated by alternative RNA splicing [17, 18]. To determine the existence of secreted CD74-spliced variants lacking transmembrane, we used RNA sequencing. Three melanoma cell lines and THP-1 MΦ were treated with IFN-γ or left untreated as a control, then subjected to total RNA extraction. All observed transcripts for CD74 are listed in Supplementary Fig. 4A and B, including coding and noncoding variants. Among coding proteins, we found that two transcripts corresponding to the p41 and p43 isoforms (transcript ENST00000009530) and the p33 and p35 isoforms (transcript ENST00000353334) were elevated under IFN-γ-stimulated conditions in A375, SK-MEL-2, and THP-1 MΦ, in agreement with WB data (Fig. 2A). However, new secreted CD74 variants were not discovered by our approach in this study.

Collectively, these results suggested that sCD74 was produced by ADAM10- and ADAM17-mediated cell-surface cleavage in SK-MEL-2 and THP-1 MΦ and by cysteine-protease-mediated lysosomal cleavage in A375.

### sCD74 suppressed tumor-cell growth by inhibiting the MIF/CD74/AKT signaling pathway under IFN-γ-stimulated conditions

**Fig. 4:**
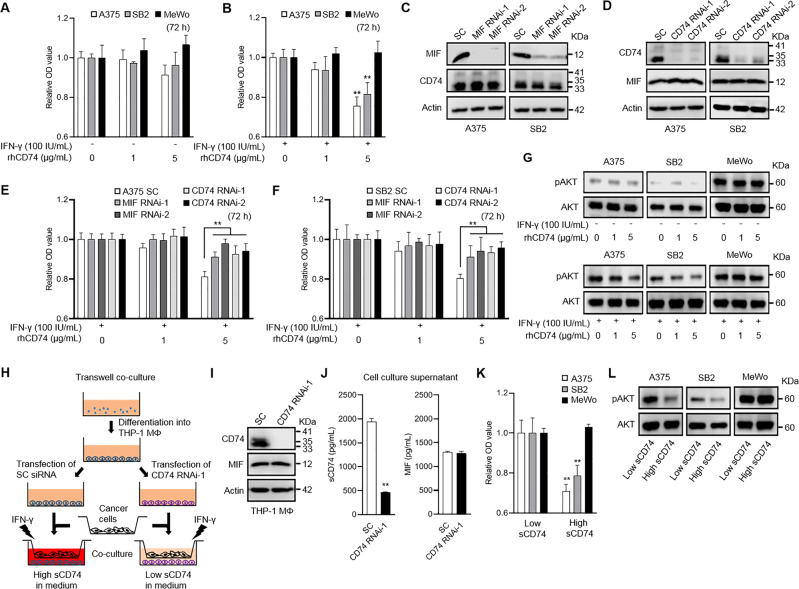
Impact of sCD74 on melanoma cell growth. **A**, **B** Cell-proliferation assay in A375, SB2, and MeWo. Cells were treated with different concentrations of rhCD74 (0, 1, and 5 µg/mL) for 72 h under basal conditions (**A**) or under 100 IU/mL IFN-γ stimulatory conditions (**B**). Results represent the fold change relative to the O.D. value of each cell line treated with 0 µg/mL rhCD74 (*n* = 6). **C** Efficacies of two individual siRNAs in knocking down MIF were analyzed by WB in A375 and SB2. MIF siRNAs did not change CD74 expression. SC siRNA was used as a reference control. **D** Efficacies of two individual siRNAs in knocking down CD74 were analyzed by WB in A375 and SB2. CD74 siRNAs did not change MIF expression. SC siRNA was used as a reference control. **E**, **F** Cell-proliferation assay in A375 (**E**) and SB2 (**F**) transfected with SC siRNA, MIF RNAi-1 and -2, and CD74 RNAi-1 and -2. Cells were treated with different concentrations of rhCD74 (0, 1, and 5 µg/mL) for 72 h under 100 IU/mL IFN-γ stimulatory conditions. Results represent the fold change relative to the O.D. value of each transfected cell treated with 0 µg/mL rhCD74 (*n* = 6). Cell-growth inhibitory effect of 5 µg/mL rhCD74 was significantly diminished in A375 and SB2 transfected with MIF RNAi-1 and -2, and CD74 RNAi-1 and -2, compared with those transfected with SC siRNA. **G** WB analysis of pAKT in A375, SB2, and MeWo treated with different concentrations of rhCD74 (0, 1, and 5 µg/mL) without IFN-γ stimulation (upper) or with 100 IU/mL IFN-γ stimulation (lower). AKT was used as a loading control. **H** Schematic illustration of transwell coculture system. **I** WB analysis of CD74 and MIF in cell lysate of THP-1 MΦ transfected with SC siRNA or CD74 RNAi-1. **J** sCD74 and MIF levels in supernatants of THP-1 MΦ transfected with SC siRNA or CD74 RNAi-1. **K** Cell-proliferation assay in A375, SB2, and MeWo 48 h after coculture with THP-1 MΦ. High and low concentrations of sCD74 in medium were obtained by transfecting SC siRNA or CD74 RNAi-1 to THP-1 MΦ, respectively, in the presence of 100 IU/mL IFN-γ. Results represent the fold change relative to the O.D. value of each cell line cultured in low sCD74-containing medium (*n* = 4). **L** WB analysis of pAKT in A375, SB2, and MeWo in low and high sCD74-containing medium. AKT was used as a loading control. Graph values represent mean ± SD. Significance in difference between two groups was tested by Student *t*-test. **p < 0.01. IFN-γ interferon-γ, MIF macrophage-migration inhibitory factor, MΦ macrophage, rh recombinant human*,* SC scramble, SD standard deviation, siRNA short-interference RNA, WB Western blot.

The survival analysis in stage-III melanoma patients directed the question of whether sCD74 is a surrogate for the CD74-expressing tumor tissues or if sCD74, per se, features some biological activities regulating tumor behaviors. To explore the impact of sCD74 on tumor growth, we first treated A375, SB2, SK-MEL-2, and MeWo with bioactive recombinant human (rh)CD74^73–232^ and investigated cell proliferation. Under basal conditions, rhCD74 did not change cell growth at any concentrations after 48 and 72 h of incubation in A375, SB2, and MeWo or after 144 h of incubation in SK-MEL-2 (Fig. 4A and Supplementary Fig. 5A, B); however, 5 μg/mL rhCD74 significantly inhibited cell growth in A375, SB2, and SK-MEL-2 under IFN-γ-stimulated conditions (Fig. 4B and Supplementary Fig. 5C, D). When A375, SB2, and MeWo were treated with a MIF enzymatic antagonist, 4-iodo-6-phenylpyrimidine (4-IPP), cell growth was suppressed in all cell lines independently of IFN-γ stimulation (Supplementary Fig. 5E, F).

Next, we explored the possible survival-signaling pathway associated with the ability of rhCD74 to inhibit cell growth in A375, SB2, and MeWo under IFN-γ-stimulated conditions. Our clinical analyses representing the ratio of sCD74 to MIF as predictive of patient survival indicated that sCD74 might interact with MIF. Besides, MIF has been proven to initiate cell-survival signaling by binding the CD74/CD44 complex, CXCR2, CXCR4, or CXCR7 [23–25]. Given that IFN-γ upregulated the expression of CD74 and pAKT, but did not alter the expression of CD44, CXCR2, CXCR4, CXCR7, MIF, or pERK1/2 in A375, SB2 (Fig. 2A, C and Supplementary Fig. 2C), we hypothesized that rhCD74 can inhibit a MIF/CD74/AKT-mediated pathway by competitively binding to MIF under IFN-γ-stimulated conditions. To test this hypothesis, we silenced MIF and CD74 genes via siRNA in A375 and SB2 (Fig. 4C, D). Silencing MIF reduced MIF release in supernatants but did not change CD74 expression (Supplementary Fig. 5G and Fig. 4C). Silencing CD74 reduced cell-surface CD74 expression (Supplementary Fig. 5H) but did not change MIF expression and MIF release in supernatants (Fig. 4D and Supplementary Fig. 5I). As expected, silencing either CD74 or MIF negated the function of rhCD74 after 72 h of incubation in A375 and SB2 (Fig. 4E, F). In addition, 5 μg/mL rhCD74 reduced the amount of pAKT signaling only under IFN-γ-stimulated conditions in A375 and SB2 (Fig. 4G). Next, to verify our hypothesis, we treated A375, SB2, and MeWo with 100 ng/mL rhMIF. Under basal conditions, rhMIF as well as any concentrations of rhCD74 did not influence cell proliferation in all cell lines; however, under IFN-γ-stimulated conditions, rhMIF promoted cell growth, which was attenuated by 5 μg/mL rhCD74 in A375 and SB2 (Supplementary Fig. 6A, B). Silencing cell-surface CD74 via siRNA diminished the proliferative activity of rhMIF and rhCD74 in A375 and SB2 under IFN-γ stimulatory conditions (Supplementary Fig. 6C, D). In addition, rhMIF enhanced pAKT signaling, which was reduced by 5 μg/mL rhCD74 only under IFN-γ-stimulated conditions in A375 and SB2 (Supplementary Fig. 6E).

Furthermore, to determine if endogenous sCD74 influences tumor progression, we developed a transwell coculture system (Fig. 4H). Two different concentrations of sCD74 and similar MIF levels in medium were obtained by transfecting SC siRNA or CD74 RNAi-1 to THP-1 MΦ in the presence of 100 IU/mL IFN-γ (Fig. 4I, J). Then, cell proliferation in A375, SB2, and MeWo was evaluated 48 h after coculture. High sCD74 in the medium released from THP-1 MΦ significantly slowed cell growth in A375 and SB2, whereas no change was observed in MeWo (Fig. 4K). Similar to the results of the rhCD74-stimulation assay, pAKT signaling was attenuated when A375 and SB2 were incubated in medium containing high sCD74 released from THP-1 MΦ (Fig. 4L). Mechanically, the inhibition of MIF activity with 4-IPP, or silencing of cell-surface CD74 by siRNA, in A375 and SB2, attenuated the antiproliferative effect of sCD74 released from THP-1 MΦ (Supplementary Fig. 7A, B), indicating that tumor-cell growth might be regulated by sCD74 released from THP-1 MΦ via modulation of MIF–CD74 interaction.

### sCD74 induced apoptosis by inhibiting MIF–CD74 signaling

**Fig. 5:**
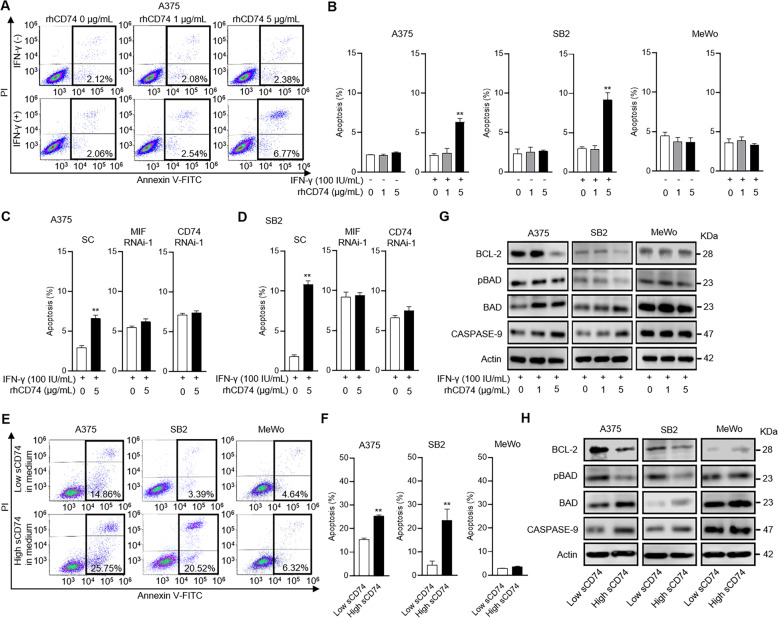
sCD74 exerts pro-apoptotic functions by inhibiting MIF–CD74 interaction. **A** Representative flow-cytometry plots show annexin V–FITC (*x* axis) and PI (*y* axis) in A375. **B** The rate of apoptotic cells in A375, SB2, and MeWo quantified by flow cytometry (*n* = 3). **C**, **D** The rate of apoptotic cells in A375 (**C**) and SB2 (**D**) transfected with SC siRNA, MIF RNAi-1, or CD74 RNAi-1 quantified by flow cytometry (*n* = 3). Flow cytometry (**A**–**D**) was performed 72 h after the administration of 0 or 5 µg/mL rhCD74 under 100 IU/mL IFN-γ stimulation. **E** Representative flow-cytometry plots show annexin V–FITC (*x* axis) and PI (*y* axis) in A375, SB2, and MeWo. **F** Rate of apoptotic cells in A375, SB2, and MeWo (*n* = 3). Flow cytometry (**E**, **F**) was performed 48 h after coculture with THP-1 MΦ. High and low concentrations of sCD74 in medium were obtained by transfecting SC siRNA or CD74 RNAi-1 to THP-1 MΦ, respectively, in the presence of 100 U/mL IFN-γ. **G** WB analysis of BCL-2, pBAD, BAD, and CASPASE-9 in A375, SB2, and MeWo 72 h after treatment with different concentrations of rhCD74 (0, 1, and 5 µg/mL) under 100 IU/mL IFN-γ stimulation. Actin and BAD were used as loading controls. **H** WB analysis of BCL-2, pBAD, BAD, and CASPASE-9 in A375, SB2, and MeWo 48 h after coculture with THP-1 MΦ. Actin and BAD were used as loading controls. Graph values represent mean ± SD. Significance in difference between two groups was tested by Student *t*-test. **p < 0.01. IFN-γ interferon-γ, MIF macrophage-migration inhibitory factor, MΦ macrophage, PI propidium iodide, rh recombinant human*,* SC scramble, SD standard deviation, siRNA short-interference RNA, WB Western blot.

Last, we explored the mechanisms by which rhCD74 or endogenous sCD74 inhibited cell growth in melanoma. Our previous investigation [5] in which autocrine MIF–CD74 signaling modulated BCL-2 expression hypothesized that sCD74 suppresses cell growth by inducing apoptosis. We therefore investigated the role of sCD74 in apoptosis quantified by flow cytometry. Under basal conditions, rhCD74 did not induce apoptosis at any concentrations in A375, SB2, or MeWo; however, 5 μg/mL rhCD74 significantly induced apoptosis only in A375 and SB2 under IFN-γ-stimulated conditions (Fig. 5A, B). This pro-apoptotic effect of rhCD74 was absent when MIF or CD74 was silenced by siRNA in A375 and SB2 (Fig. 5C, D). In addition, treatment with 5 μg/mL rhCD74 for 48 h significantly increased MIF release in supernatants of A375 and SB2, which may be associated with the induction of apoptosis by rhCD74 (Supplementary Fig. 8). Likewise, in coculture experiments with THP-1 MΦ, the rate of apoptotic cells was significantly higher in A375 and SB2 in the presence of high sCD74-containing medium compared with low sCD74-containing medium (Fig. 5E, F). In addition, a series of changes in pro-apoptosis-related proteins, including downregulation of BCL-2 expression and upregulation of the ratio of BAD to pBAD and CASPASE-9 expression, were observed in A375 and SB2 upon treatment with 5 μg/mL rhCD74 under IFN-γ-stimulated conditions (Fig. 5G), as well as upon incubation in high sCD74-containing medium (Fig. 5H).

## Discussion

The interference of the soluble form of cytokine receptors in the cytokine-receptor axis yields the complexity of cytokine signaling and sequential inflammation, which may in turn determine cancer development and progression [14, 26]. Numerous studies, therefore, have assessed the potentiality of soluble forms of receptors as diagnostic and survival predictors using patients’ blood samples [17, 18, 27–30]; in fact, serum-soluble interleukin-2 receptor has been widely utilized as an adjunct to the diagnosis and a response evaluation of malignant lymphoma in the clinical setting [30]. In the current study, the combination of sCD74 and MIF was a significant survival predictor for melanoma patients with advanced stage. It is of substantial interest that these two partnering proteins in sera helped stratify patient survival in the same way as those in the tumor [6], although the underlying biological meanings may be different. Our findings suggest that serum sCD74 will not be suitable for diagnostic purposes, as its levels were similar between healthy donors and early-stage melanoma patients. However, sCD74 released by tumor cells or tumor-evoked surrounding cells may contribute to the elevated level of serum sCD74 in advanced melanoma. Indeed, this mechanism seems plausible based on our previous observations that approximately 80% of patients with advanced melanoma were positive for CD74 in the tumor and tumor-infiltrating immune cells [5, 6]. Our in vitro experiments validated that sCD74 was predominantly secreted by the majority of melanoma cell lines and THP-1 MΦ, especially in response to IFN-γ. On the contrary, sCD74 release from other immune cells was unexpectedly low to absent. However, we did not evaluate sCD74 release from the activated immune cells specific to the tumor. Further investigations of immune-cell-derived sCD74 would provide new insight into how sCD74 influences tumor behaviors and the tumor microenvironment.

The composition of sCD74 has not been well documented yet. Only a single study [21] has assessed the sCD74 components using serum samples of autoimmune liver-disease patients, and a single 25-KDa band was detected by immunoprecipitation followed by an immunoblot assay. In agreement, our study detected a band of the same size in sera of melanoma patients and healthy donors. Importantly, the intensity of the 25-KDa bands was positively correlated with serum sCD74 levels quantified by ELISA, indicating this band’s clinical relevance. However, our serum samples also contained two additional bands of 18 KDa and 15 KDa, the former particularly clearer. Although these bands may be originated from the cells that we did not target in this study or from one of the degradation fragments of CD74, the 18-KDa bands were more frequently detected in the sera of melanoma patients than NHDs (data not shown), suggesting that it may participate in tumor-progression events. Further analysis by mass spectrometry in future studies would help us gain more knowledge of sCD74 components in sera.

The production mechanisms of the soluble form of receptors will inform not only disease pathophysiology but also the development of potential therapeutic drugs [14]. Research has identified specific sheddases for CD74 during generation of CLIP and CD74 cytosolic intracellular domains (ICDs), as well as N-terminal fragments (NTFs) in the endosomal membrane [31, 32]. The sCD74 production by cysteine-protease-mediated cleavage may be accompanied by these processes. However, an inhibitor targeting cathepsin S, which we thought was a highly possible cysteine protease, did not suppress sCD74 release in supernatants in A375. Further investigation is needed to determine the specific cysteine protease involved in sCD74 production. In addition, regulated intramembrane proteolysis (RIP) of endosomal CD74 by signal-peptide peptidase-like 2A (SPPL2A) is indispensable for B-cell development [32], and a previous study [21] showed that sCD74 was produced by RIP-based cleavage; however, this cleavage is followed by ectodomain canonical cleavage and is less likely to be directly associated with sCD74 production. Indeed, knockdown of SPPL2A and SPPL2B did not change the release of sCD74 in supernatants in our series (data not shown). In contrast, ADAM10 and ADAM17 have been identified as canonical sheddases prior to RIP-based cleavage for some type-II transmembrane proteins, including TNF-α and Fas ligand [33, 34], and the same may be true for CD74.

The soluble form of receptors can modulate signaling activity in either an agonistic or antagonistic fashion, depending on the nature and structure of its original receptors and ligands [14]. As for sCD74, Leng et al. addressed that 5 µg/mL or higher recombinant CD74^73–232^ inhibited MIF recognition and real-time binding analysis determined nM affinity binding of MIF to CD74^73–232^ [9]. Assis et al. also reported that serum sCD74 from patients with autoimmune liver disease neutralized MIF bioactivity, thereby inactivating ERK signaling [21]. Moreover, Soppert et al. reported that sCD74 induced the necrosis of murine cardiac myofibroblasts by hampering MIF/CXCR4/AKT-mediated survival [35]. Our present work suggests that sCD74 displayed moderate antiproliferative capacity, by acting as a “decoy” receptor for MIF, and was antagonistic to the MIF/CD74/AKT-survival signaling pathway in melanoma. Notably, our finding that sCD74, unlike an MIF enzymatic inhibitor, elicited antisurvival and pro-apoptotic effects specific for cell-surface CD74-expressing tumor cells induced by IFN-γ indicates that sCD74 may not interfere with other receptor-dependent MIF signaling or receptor-independent endocytotic MIF signaling. Although MIF-binding sites for its receptors remain under investigation [36, 37], a recent study showed that a peptide designed to mimic the CXCR4-binding site to MIF selectively blocked the MIF/CXCR4 axis without interfering the MIF/CD74 axis, which would support our results [38]. In addition, our coculture assay indicated that M1-like macrophages may remotely contribute to suppressing tumor-cell growth though the release of sCD74.

This study has several limitations. First, this was a retrospective observational study and the sample size was small. A prospective study with larger sample size is necessary to validate the impact of serum sCD74 and MIF levels on melanoma-patient survival. Besides, we could not show the significant association between CD74 and MIF expressions in tumor tissues, including tumor-infiltrating immune cells and sCD74 and MIF levels in paired sera, respectively. Evaluation of these associations with larger cohorts would help identify the potential cellular sources responsible for circulating sCD74 and MIF in melanoma. Second, serum samples at different stages or at different treatment time points in each patient were unavailable due to the retrospective nature of this study. Assessment of changes in circulating these two proteins may not only be useful as a prediction of treatment outcome but also informs the potential cellular sources of these proteins in melanoma. Third, we could not account for the reason why sCD74 production mechanisms differed across cell types. Finally, we did not exhibit the roles of sCD74 and MIF interaction in TIME. Our functional study indicates that the activity of this interaction largely depends on the presence of IFN-γ produced by immune cells in TIME. Further investigation focusing on TIME would provide a better understanding of the impact of this interaction on tumor behaviors.

In conclusion, the present study demonstrated the distribution and survival significance of serum sCD74 in patients with melanoma. We also showed the composition and production mechanisms, as well as the biological aspect, of sCD74 interacting with MIF. We propose that full-length CD74 and various fragments of CD74 (CLIP, CD74–ICDs, CD74–NTFs, and sCD74) from different isoforms may carry out distinct functions. In this regard, distinct expression patterns of these CD74 forms may predispose patients to substantial differences in cancer progression and survival. Furthermore, with a better understanding of these regulatory mechanisms, the beneficial modulation of these CD74 forms may lead to therapeutic approaches.

## Supplementary information

Supplementary Appendix

Supplementary Figures

Supplementary Tables

## Data Availability

The data that support the findings of this study are available from the corresponding author upon reasonable request.
