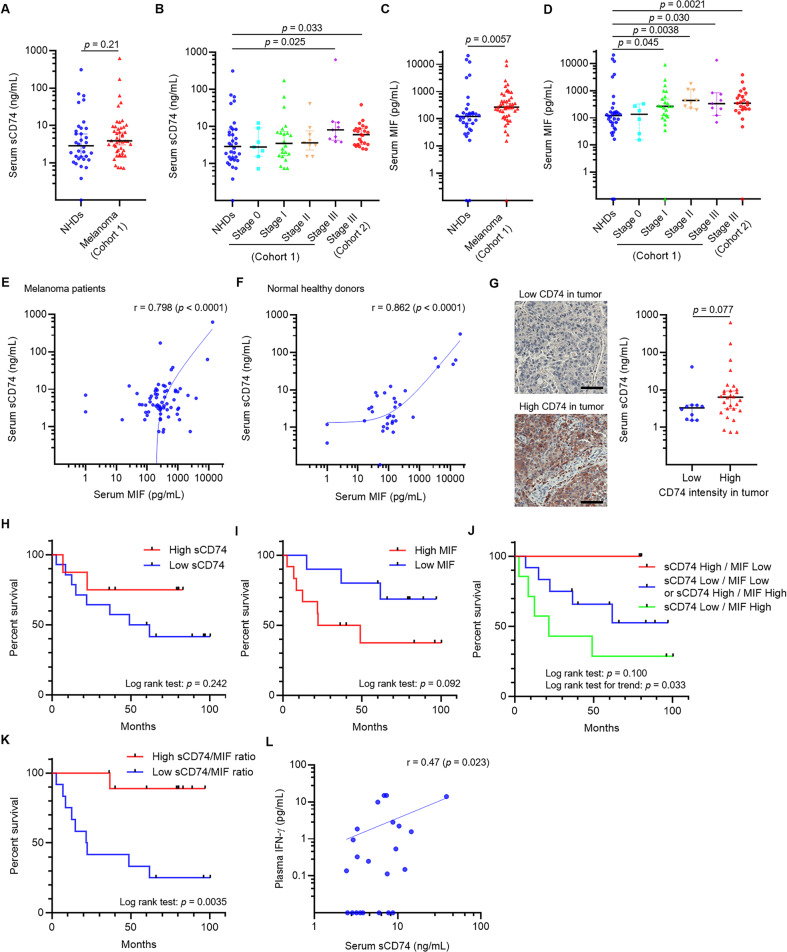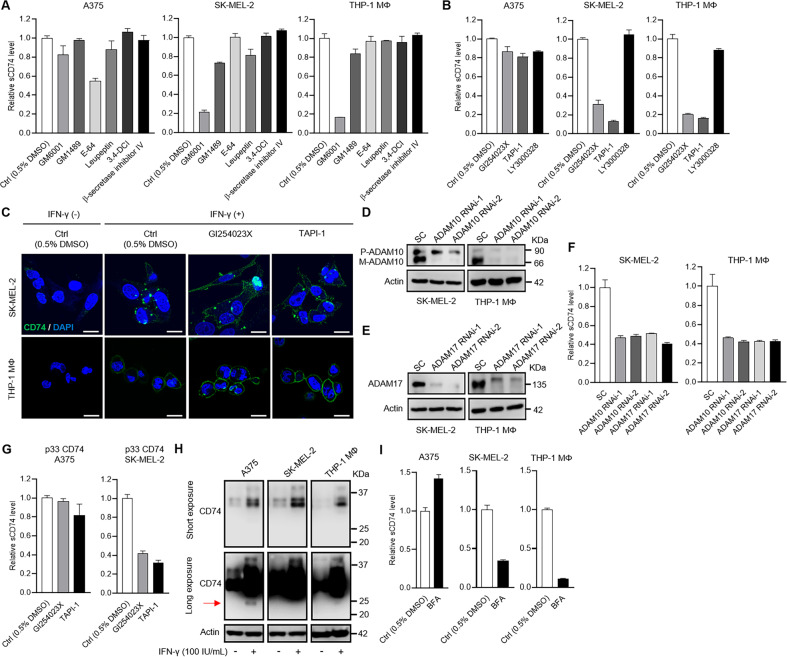# Correction: Interplay between soluble CD74 and macrophage-migration inhibitory factor drives tumor growth and influences patient survival in melanoma

**DOI:** 10.1038/s41419-022-04879-6

**Published:** 2022-05-02

**Authors:** Yasunari Fukuda, Matias A. Bustos, Sung-Nam Cho, Jason Roszik, Suyeon Ryu, Victor M. Lopez, Jared K. Burks, Jeffrey E. Lee, Elizabeth A. Grimm, Dave S. B. Hoon, Suhendan Ekmekcioglu

**Affiliations:** 1 Department of Melanoma Medical Oncology, The University of Texas MD Anderson Cancer Center, Houston, TX 77030 USA; 2 Department of Translational Molecular Medicine, Saint John’s Cancer Institute, Providence Saint John’s Health Center, Santa Monica, CA 90404 USA; 3 Department of Genomic Medicine, The University of Texas MD Anderson Cancer Center, Houston, TX 77030 USA; 4 Department of Genome Sequencing, Saint John’s Cancer Institute, Providence Saint John’s Health Center, Santa Monica, CA 90404 USA; 5 Department of Leukemia, The University of Texas MD Anderson Cancer Center, Houston, TX 77030 USA; 6 Department of Surgical Oncology, The University of Texas MD Anderson Cancer Center, Houston, TX 77030 USA

**Keywords:** Melanoma, Prognostic markers

Correction to: *Cell Death & Disease* 10.1038/s41419-022-04552-y published online 04 February 2022

The original version of this article unfortunately contained mistakes. Most unfortunately, after the publication of their manuscript, the authors noticed that Fig. 1B and D, Fig. 3D and H were incorrect. In Fig. 1B and D, the labels on the x-axis were correct, but graphs themselves of Fig. 1B and D were inversely displayed. Only graphs themselves moved -all axis titles and *p* values bars were in correct places. In Fig. 3D, actin band of THP-1 MΦ was in an accidental duplicate of that of THP-1Φ in Fig. 3E. Replaced with correct actin band image in Fig. 3D In Fig. 3H, actin band of THP-1 MΦ was in an accidental duplicate of that of SK-MEL-2. Replaced with correct actin band image in Fig. 3H, THP-1 MΦ panel. The authors declare that these corrections do not change the results or conclusions of their paper. The corrected figures can be found below. The original article has been corrected.