# Supplementary material for: Interplay between soluble CD74 and macrophage-migration inhibitory factor drives tumor growth and influences patient survival in melanoma
Source: Cell Death Dis. 2022 Feb 4;13(2):117. doi: 10.1038/s41419-022-04552-y (PMC8816905; doi:10.1038/s41419-022-04552-y)
Supplement: Supplementary file 1 — Supplementary Appendix [file 41419_2022_4552_MOESM1_ESM.docx]

SUPPLEMENTARY APPENDIX

**Interplay between soluble CD74 and macrophage migration inhibitory factor drives tumor growth and influences patient survival in melanoma**

**Yasunari Fukuda^1^, Matias A. Bustos^2^, Sung-Nam Cho^1^, Jason Roszik^3^, Suyeon Ryu^4^, Victor M. Lopez^4^, Jared K. Burks^5^, Jeffrey E. Lee^6^, Elizabeth A. Grimm^1^, Dave S. Hoon^4^ and Suhendan Ekmekcioglu^1^**

^1^Department of Melanoma Medical Oncology, The University of Texas MD Anderson Cancer Center, Houston, TX 77030, USA

^2^Department of Translational Molecular Medicine, Saint John’s Cancer Institute, Providence Saint John’s Health Center, Santa Monica, CA 90404, USA

^3^Department of Genomic Medicine, The University of Texas MD Anderson Cancer Center, Houston, TX 77030, USA

^4^Department of Genome Sequencing, Saint John’s Cancer Institute, Providence Saint John’s Health Center, Santa Monica, CA 90404, USA

^5^Department of Leukemia, The University of Texas MD Anderson Cancer Center, Houston, TX 77030, USA

^6^Department of Surgical Oncology, The University of Texas MD Anderson Cancer Center, Houston, TX 77030, USA.

**Supplementary Appendix**

**Additional materials and methods**

**Enzyme-linked immunosorbent assay (ELISA)**

Four melanoma cell lines, THP-1 cells and primary M0 MΦ, and other primary immune cells (CD4^+^ T cells, CD8^+^ T cells, B cells and NK cells) were seeded onto 6-well plates at a density of 2×10^5^, 4×10^5^ and 2×10^6^ cells, respectively, to equalize total protein concentrations at the point of cell culture supernatant collection. After 24-h incubations (for THP-1, 24 h after the induction of differentiation into MΦ and for primary M0 MΦ, 24 h after all cells were attached on the plate), cells were placed in serum-starved medium with 1% FBS to reduce background cytokine expression and treated with different concentrations of rhIFN-γ (R&D systems; 50 IU/mL is equivalent to 10 ng/mL), or rhIL-4 and rhIL-13 (PeproTech; Rocky Hill, NJ, USA). Cells were also treated simultaneously with several protease inhibitors purchased from Cayman Chemical (Ann Arbor, MI, USA) to explore the potential proteolytic enzymes of CD74 (Supplementary Table S2). All protease inhibitors were dissolved in dimethyl sulfoxide (DMSO). After 24 h of cytokine and inhibitor application, supernatants were centrifuged at 3000*g* for 10 min and collected for subsequent assays. sCD74, MIF, and IFN-γ levels were measured using ELISA kits according to manufacturer recommendations in serum and plasma samples as well as cell culture supernatants. A Human CD74 ELISA Kit was purchased from Sigma-Aldrich (St. Louis, MO, USA; immunogens for both capture and detection polyclonal Abs are 73-232 aa of CD74); a Human MIF DuoSet ELISA Kit was purchased from R&D Systems (Minneapolis, MN, USA); and an IFN gamma Human ELISA Kit (High Sensitivity) was purchased from Invitrogen (Carlsbad, CA, USA).

**Western blot (WB) analysis**

Total proteins were extracted with RIPA buffer, supplemented with [1% protease inhibitor cocktail](https://www.thermofisher.com/order/catalog/product/78420) and [1% phosphatase inhibitor cocktail](https://www.thermofisher.com/order/catalog/product/78420) (Thermo Fisher Scientific, Waltham, MA, USA). Protein aliquots were electrophoresed on either Novex WedgeWell 4%-20% gel for CD44, CXCR2, CXCR4, CXCR7, CD74, ADAM10, ADAM17, CASPASE-9, and Actin), 4%-12% gel for pAKT, AKT, pERK1/2, and ERK1/2, or 16% gel for MIF, BCL-2, pBAD and BAD (Thermo Fisher Scientific), followed by transfer to nitrocellulose membranes. Loading samples and doses (20 µg) were definitely same in each experiment in case we used different gels. After blocking with either 5% nonfat milk or BSA for 1 h at room temperature (RT), membranes were incubated overnight at 4°C with primary Abs, as listed in Supplementary Table S3. Subsequently, membranes were incubated with IgG HRP linked secondary Abs (1:1000; R&D Systems) for 1 h at RT. Enzymatic signals were visualized with the SuperSignal West Pico Chemiluminescent Substrate (Thermo Fisher Scientific). Restore Western Blot Stripping Buffer (Thermo Scientific) was used to remove the primary and secondary Abs, if applicable.

**Deglycosylation reaction**

The Protein Deglycosylation Mix II (New England BioLabs Inc., Ipswich, MA, USA) was used to evaluate the glycosylation status of sCD74 in cell culture supernatants or patient’s serum, according to manufacturer recommendations. Briefly, 25 μL Deglycosylation Mix Buffer 2 was added into 200 μL samples, followed by incubation for 10 min at 72°C$.$ After cooldown, 5 μL Protein Deglycosylation Mix II was applied and incubated for 30 min at RT (for negative control, 5 μL phosphate-buffered saline was added, instead)$.$ Subsequently, samples were incubated for another 1 h at 37°C. Deglycosylated proteins were concentrated using acetone precipitation and subjected to WB.

**Acetone Precipitation**

Acetone precipitation was performed to obtain the maximum possible amount of sCD74 proteins in supernatants or patient’s serum. Briefly, cell culture supernatants or patient’s serum were concentrated using Amicon Ultra-4 Centrifugal Filter Units (MilliporeSigma, Burlington, MA, USA). Then, four times volume of cold acetone was applied into concentrated samples and incubated for 3 h at −20°C, followed by centrifugation at 14000*g* for 10 min. Protein pellets were dissolved in reducing sample buffer and subjected to WB.

**Short interfering RNA (siRNA)-mediated gene knockdown**

ON-TARGETplus siRNA reagents targeting two different sequences of ADAM10, ADAM17, CD74, and MIF (GE Healthcare Dharmacon Inc., Lafayette, CO, USA; target sequences are listed in Supplementary Table S4) were used to suppress expression of these gene via siRNA (referred to by the gene name followed by “RNAi-1” and “RNAi-2”, hereafter). Scramble (SC) siRNA (Dharmacon Inc.) was also applied as a negative control. Each cell was transfected with 20 nM siRNA reagents using lipofectamine RNAiMAX (Invitrogen) according to the manufacturer's recommendations. Transfection to the THP-1 cell line was performed after the induction of differentiation into MΦ. Efficacy of transfection was confirmed by real-time polymerase chain reaction (data not shown) and WB.

**CD74 isoforms stable overexpression**

We outsourced the generation of three lentivirus particles to VectorBuilder (Chicago, IL, USA): the control lentivirus packaging pLV[Exp]-EGFP:T2A:Puro-EF1A>mCherry as well as human CD74 p33 and p35 isoform lentivirus packaging pLV[Exp]-EGFP:T2A:Puro-EF1A>{hCD74-p33 isoform} and {hCD74-p35 isoform}, respectively. The CD74-encording lentivirus particles were transduced into a 40% confluent cell lines with 5 µg/mL polybrene. Transduced cells were selected with puromycin. Efficacy of transfection was confirmed by WB.

**Cell proliferation assay**

Cell proliferation was measured by two protocols. In the first protocol, melanoma cell lines were seeded onto 96-well plates at a density of 4×10^3^ cells/well and exposed to different concentrations of rhCD74 (Novus Biologicals, Littleton, CO, USA), 4-iodo-6-phenylpyrimidine (4-IPP, Sigma-Aldrich), or rhMIF (PeproTech) with/without 100 IU/mL IFN-γ stimulation. After 48 and 72 h of culture (for A375, SB2, and MeWo) and 144 h of culture (for SK-MEL-2), cells were incubated with PrestoBlue Cell Viability Reagent (Thermo Fisher Scientific) for 2 h according to the manufacturer’s recommendations. The absorbance was monitored with a spectrophotometer at 570 nm using 600 nm as a reference wavelength. In the second protocol, THP-1 cells were seeded onto 6-well plates at a density of 1×10^6^ cells/well and differentiated into THP-1 MΦ. THP-1 MΦ cells were transfected with SC siRNA or CD74 RNAi-1. A375, SB2, and MeWo (5×10^4^/well) were separately seeded onto a 0.4-μm porous insert layer at the same day of transfection. Two layers were combined for co-culture the next day and incubated over 48 h in the presence of 100 IU/mL IFN-γ. Then, cancer cells were incubated with 1 mg/mL 3-(4,5-dimethylthiazol-2-yl)-2,5-diphenyltetrazolium bromide (MTT; Sigma-Aldrich) solution for 3 h at 37°C. The precipitate formed was dissolved in DMSO. The absorbance was monitored with a spectrophotometer at 570 nm using 630 nm as a reference wavelength.

**Cell-surface CD74 expression**

Immunocytochemistry and flow cytometry assays were performed to examine cell-surface CD74 expression. For immunocytochemistry, cells cultured onto μ-Plate 24 Well Black (ibidi, Martinsried, Planegg, Germany) were fixed in 4% paraformaldehyde for 15 min and blocked with 1% bovine serum albumin (BSA) for 1 h at RT. Next, cells were incubated with mouse anti-CD74 mAb (M-B741) (1:200; Santa Cruz Biotechnology, Dallas, TX, USA) for 1 h at RT. Then, cells were stained with goat anti-mouse Alexa Fluor 488–conjugated IgG (H+L) high cross-absorbed secondary Ab (1:500; Thermo Fisher Scientific) for 1 h at RT. Sequentially, cells were counterstained with 4′,6-diamidino-2-phenylindole (DAPI) solution and analyzed by an FV1000 microscope (Olympus, Shinjuku, Tokyo, Japan). For flow cytometry, dispersed cells were stained with mouse anti-CD74 mAb (M-B741; BD Biosciences, Franklin Lakes, NJ, USA or LN-2; BioLegend, San Diego, CA, USA) for 30 min at 4°C and analyzed by the Gallios flow cytometer (Beckman Coulter, Brea, CA, USA).

**Apoptosis assay**

Melanoma cell lines were treated with different concentrations of rhCD74 for 72 h or co-cultured with THP-1 MΦ for 48 h. Apoptosis was assessed using Annexin V-FITC Apoptosis Kit (BioVision Inc., Milpitas, CA, USA), according to the manufacturer's recommendations, and quantified with the Gallios flow cytometer (Beckman Coulter).

**Synthesis of next-generation sequencing libraries**

RNA was isolated with the RNeasy Mini Kit (Qiagen, Hilden, Germany), according to manufacturer’s recommendations. The quality of the extracted RNA samples was examined with Agilent TapeStation digital electrophoresis (Agilent Technologies, Santa Clara, CA, USA). Only samples with a RIN ≥ 7.0 were included for further analysis. RNA samples were fragmented by using divalent cations and boiling at 94°C. From the fragmented RNA, the cDNA libraries were prepared using Illumina TruSight RNA Fusion Kit (Illumina, San Diego, CA, USA). Briefly, cDNA was generated through random priming for first and second strand synthesis followed by adapter ligation, then amplified with 15 cycles of PCR. Targeted coding regions of the CD74 gene were hybridized with sequence-specific probes. The probes were selectively captured by Streptavidin Magnetic Beads (SMB) and then washed to remove any nonspecific binding. The probe hybridization and SMB capturing steps were repeated twice to maximize the selective capture, and then bead-based purification was performed with AMPureXP beads (Beckman Coulter). Second PCR amplification was performed with 14 cycles, followed by another bead-based purification. The enriched final libraries were quantified with a Qubit 4 fluorometer (Invitrogen) and via qPCR analysis using KAPA Library Quant Kit (Illumina). Library base-pair distributions and purities were confirmed with Agilent TapeStation 2200 using the High Sensitivity D1000 Screentape and High Sensitivity D1000 reagents. All libraries were normalized and pooled together to be sequenced on the Illumina NextSeq 550 platform High Output Kit at 76 cycles.

**Bioinformatics analysis**

The generated FASTQ files for melanoma cell lines (A375, SK-MEL-2, and MeWo) and THP1 MΦ were fed into Illumina DRAGEN RNA Pipeline version 3.7.5 on BaseSpace (https://www.illumina.com/products/by-type/informatics-products/dragen-bio-itplatform). This pipeline performs secondary analysis of next-generation sequencing data using a splicing-aware aligner to detect splicing junctions and potential exons in the transcripts. Human genome hg38 was selected as the reference genome. The output of this pipeline includes BAM (.bam file extension) files alongside BAI-format BAM index files (.bai file extension) in the same directory. BAM files were uploaded on Integrative Genomics Viewer (IGV) version 2.9.3 for visualization of the CD74 gene. All images were generated using IGV and exported as PNG files. The final figures were processed using Adobe Illustrator.

CD74 mRNA expression in skin cutaneous melanoma (SKCM) and normal tissues were obtained from public The Cancer Genome Atlas (TCGA) data repositories (<http://tcga-data.nci.nih.gov> and <http://gdac.broadinstitute.org/>) and The Genotype-Tissue Expression (GTEx) Portal (<http://www.gtexportal.org>), respectively. We converted the expression value to transcripts per million (TPM) to compare the expression between melanoma and normal tissues.
