# Supplementary material for: Interplay between soluble CD74 and macrophage-migration inhibitory factor drives tumor growth and influences patient survival in melanoma
Source: Cell Death Dis. 2022 Feb 4;13(2):117. doi: 10.1038/s41419-022-04552-y (PMC8816905; doi:10.1038/s41419-022-04552-y)
Supplement: Supplementary file 2 — Supplementary Figures [file 41419_2022_4552_MOESM2_ESM.docx]

SUPPLEMENTARY FIGURES

**Interplay between soluble CD74 and macrophage migration inhibitory factor drives tumor growth and influences patient survival in melanoma**

**Yasunari Fukuda^1^, Matias A. Bustos^2^, Sung-Nam Cho^1^, Jason Roszik^3^, Suyeon Ryu^4^, Victor M. Lopez^4^, Jared K. Burks^5^, Jeffrey E. Lee^6^, Elizabeth A. Grimm^1^, Dave S. Hoon^4^ and Suhendan Ekmekcioglu^1^**

^1^Department of Melanoma Medical Oncology, The University of Texas MD Anderson Cancer Center, Houston, TX 77030, USA

^2^Department of Translational Molecular Medicine, Saint John’s Cancer Institute, Providence Saint John’s Health Center, Santa Monica, CA 90404, USA

^3^Department of Genomic Medicine, The University of Texas MD Anderson Cancer Center, Houston, TX 77030, USA

^4^Department of Genome Sequencing, Saint John’s Cancer Institute, Providence Saint John’s Health Center, Santa Monica, CA 90404, USA

^5^Department of Leukemia, The University of Texas MD Anderson Cancer Center, Houston, TX 77030, USA

^6^Department of Surgical Oncology, The University of Texas MD Anderson Cancer Center, Houston, TX 77030, USA.

**Supplementary Figures**

Supplementary Fig. 1

**
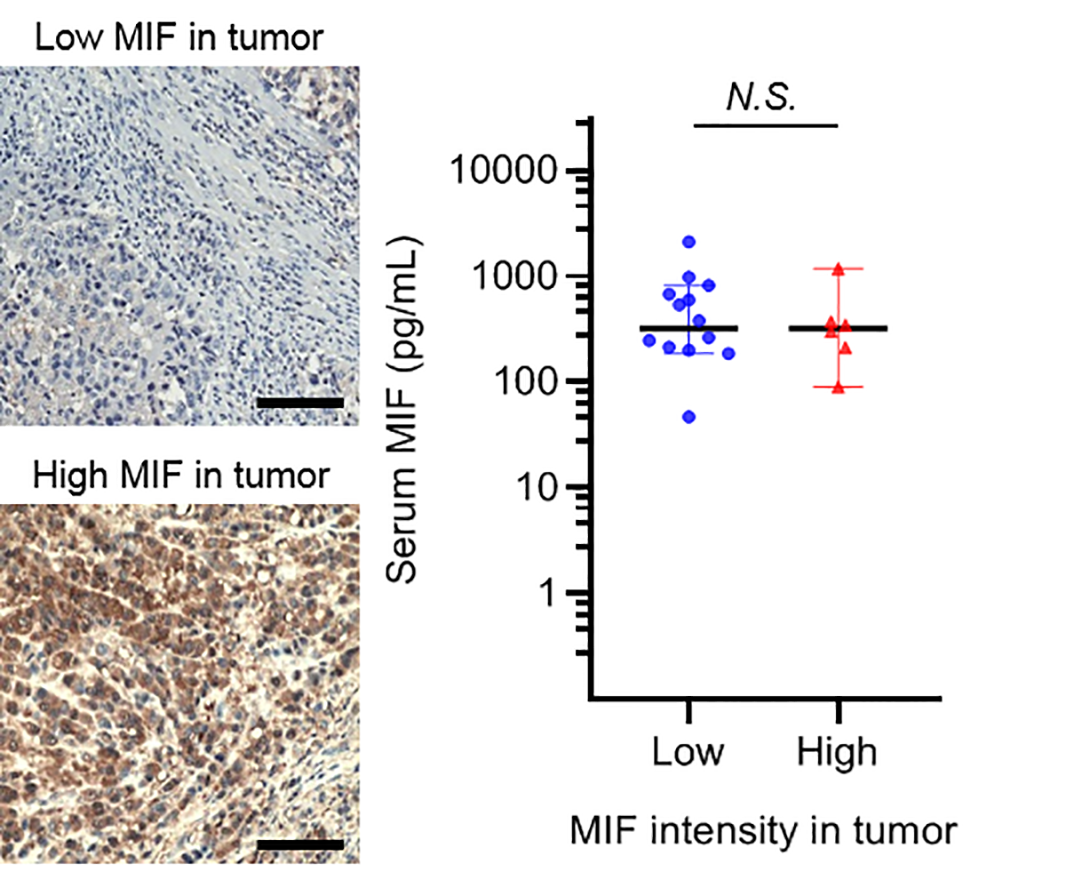
**

Supplementary Fig. 1 Association between serum MIF levels and MIF expression in tumor tissues

Representative images of low and high MIF intensity (left) in tumor.

Box plot of serum MIF levels (right) in patients with low and high MIF intensity in tumor in cohort 2. Scale bar = 100 μm.

*MIF* macrophage migration inhibitory factor, *N.S*. not significant

Supplementary Fig. 2


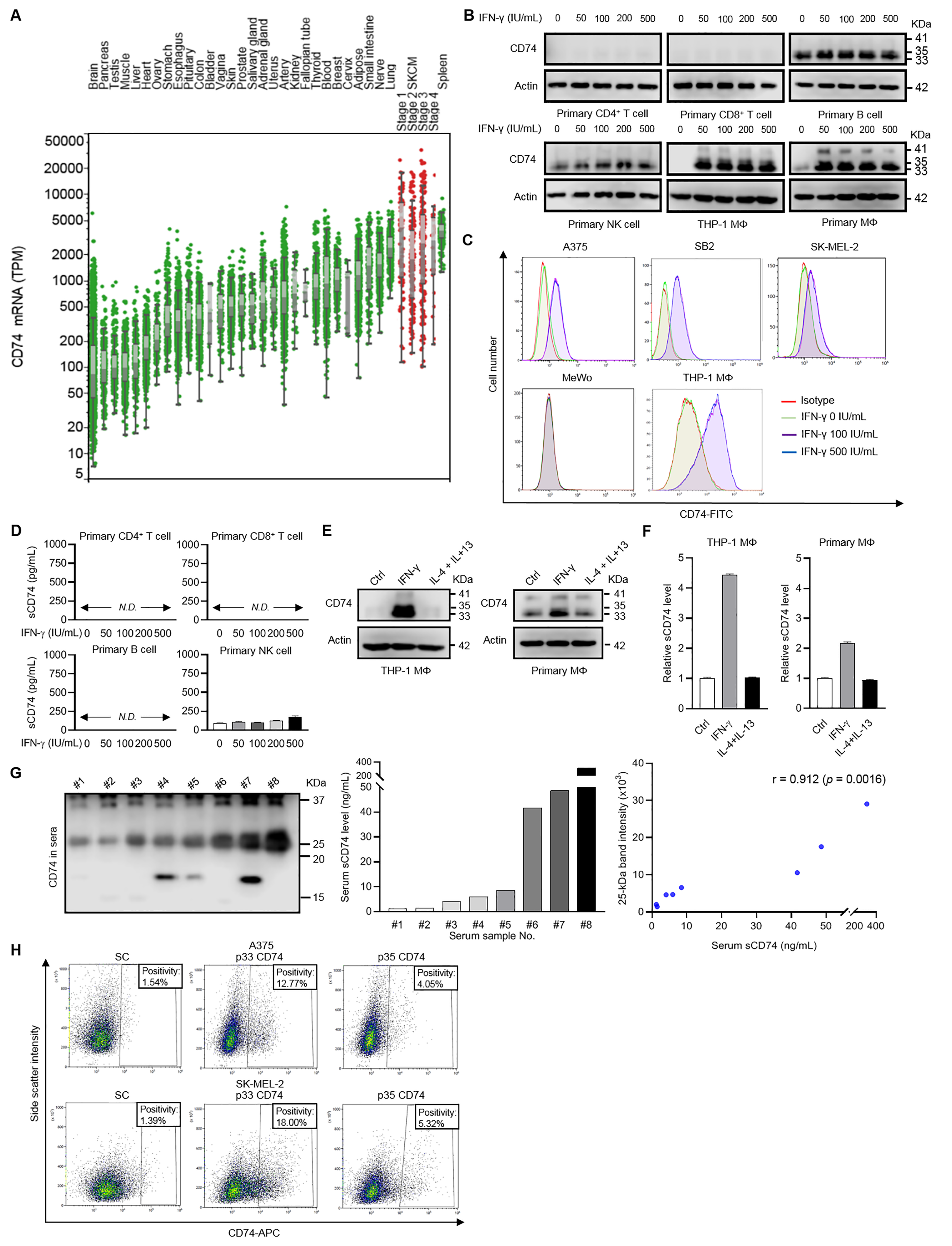


Supplementary Fig. 2 CD74 expression and release of sCD74 from melanoma cell lines and immune cells

(A) CD74 mRNA expression in normal tissues (n=2921) and SKCM tissues (n=470). The expression values are converted to TPM acquired from TCGA data repositories and GTEx portal. (B) WB analysis of changes in CD74 expression in response to IFN-γ (0 to 500 IU/mL) in primary CD4^+^ T cells, CD8^+^ T cells, B cells, NK cells, THP-1 MΦ, and primary MΦ. Actin was used as a loading control. (C) Flow cytometry assay for cell-surface CD74 expression in A375, SB2, SK-MEL-2, MeWo, and THP-1 MΦ after 0, 100, and 500 IU/mL IFN-γ stimulation. Histogram: red, isotype control; green, untreated cells stained with CD74-FITC Ab; purple, 100 IU/mL IFN-γ-treated cells stained with CD74-FITC Ab; blue, 500 IU/mL IFN-γ-treated cells stained with CD74-FITC Ab. (D) Release of sCD74 in supernatants after IFN-γ stimulation (0 to 500 IU/mL) in primary culture CD4^+^ T cells, CD8^+^ T cells, B cells, and NK cells measured by ELISA (n=3). (E) WB analysis of CD74 expression in THP-1 MΦ and primary MΦ treated with 100 IU/mL IFN-γ or combination of 20 ng/mL rhIL-4 and 20 ng/mL rhIL-13. Actin was used as a loading control. (F) Release of sCD74 in supernatants after 100 IU/mL IFN-γ stimulation or combination of 20 ng/mL rhIL-4 and 20 ng/mL rhIL-13 in THP-1 MΦ and primary MΦ measured by ELISA (n=3), and the fold-change relative to sCD74 levels in supernatants of ctrl cells is shown as bar graphs. (G) WB analysis of sCD74 in sera (upper) and serum sCD74 levels measured by ELISA (middle) in corresponding samples (n=8). Correlation between the intensity of 25-kDa sCD74 bands detected by WB and serum sCD74 levels (below). The intensity of WB bands was quantified using Image J (National Institutes of Health, USA). (H) Flow cytometry assay for cell-surface CD74 expression in A375 and SK-MEL-2 after CD74 overexpression. SC, p33 CD74, and p35 CD74 overexpressed cells were stained with isotype control or CD74-APC Ab. Graph values represent mean ± SD. The Pearson correlation coefficient was used to measure the strength of a linear association between serum sCD74 levels and 25-kDa band intensity.

*ELISA* enzyme-linked immunosorbent assay, *GTEx* Genotype-Tissue Expression, *IFN-γ* interferon-γ,

*MΦ* macrophage, *NK* natural killer, *rh* recombinant human, *SD* standard deviation, *SKCM* skin cutaneous melanoma, *TCGA* the Cancer Genome Atlas, *TPM* transcripts per million, *WB* Western blot

Supplementary Fig. 3


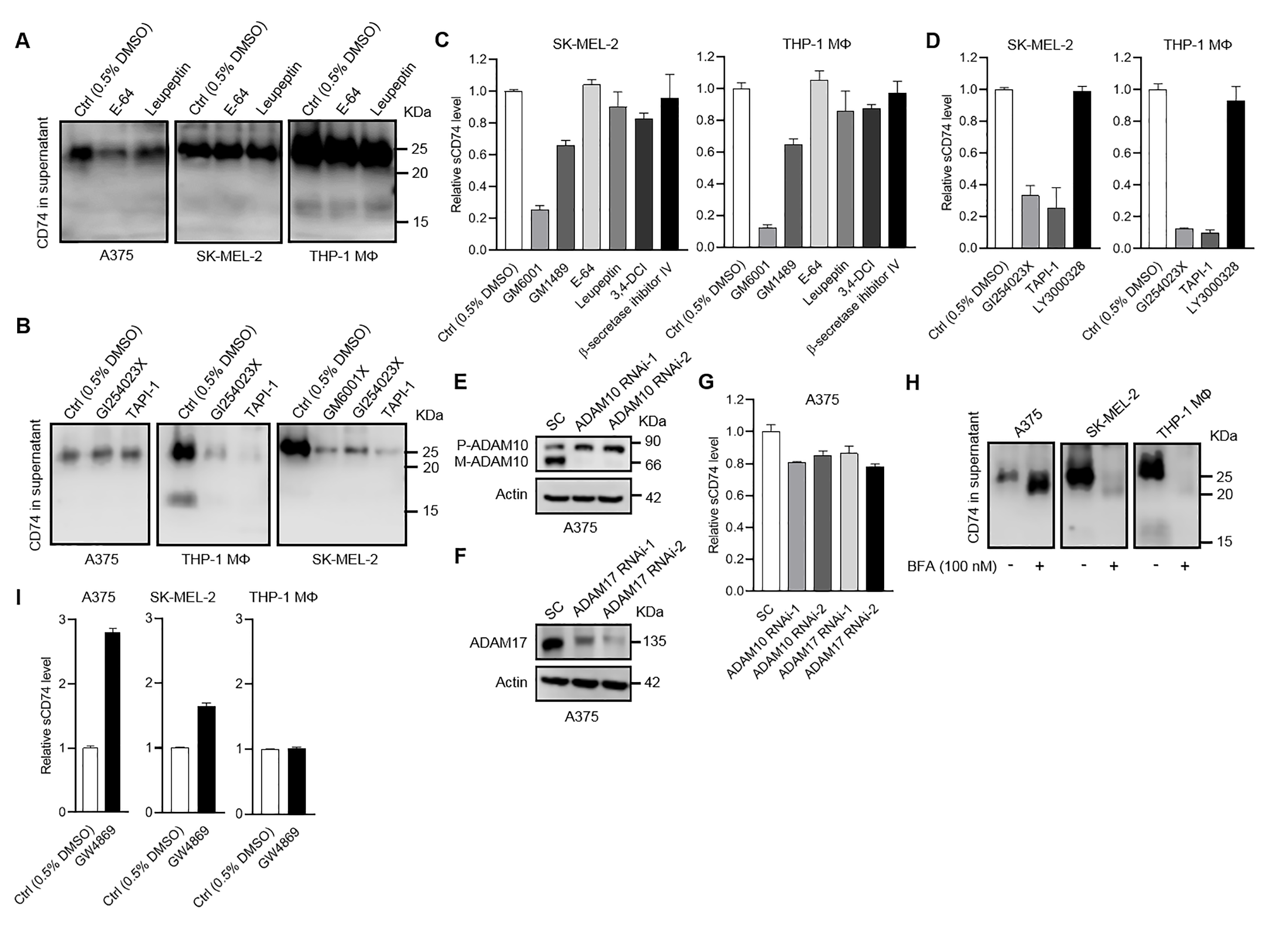
Supplementary Fig. 3 Protease inhibitory assay in melanoma cells and THP-1 derived macrophages

(A,B) WB analysis of sCD74 in supernatants of A375, THP-1 MΦ, and SK-MEL-2 after 24 h exposure to E64 and leupeptin (A), or GI254023X, TAPI-1, and GM6001 (B) under 500 IU/mL IFN-γ stimulatory conditions. (C,D) SK-MEL-2 and THP-1 MΦ were treated with GM6001, GM1489, E-64, Leupeptin, 3,4-DCI, and β-secretase inhibitor IV (C), or GI254023X, TAPI-1, and LY3000328 (D) for 24 h under basal conditions. Release of sCD74 in supernatants was measured by ELISA (n=3) and the fold-change relative to sCD74 levels in supernatants of cells treated with 0.5% DMSO are shown as bar graphs. (E) Efficacies of two individual siRNAs in knocking down ADAM10 expression were analyzed by WB in A375. SC siRNA was used as a reference control. (F) Efficacies of two individual siRNAs in knocking down ADAM17 expression were analyzed by WB in A375. SC siRNA was used as a reference control. (G) Release of sCD74 in supernatants was measured by ELISA in A375 transfected with SC siRNA, ADAM10 RNAi-1, -2 and ADAM17 RNAi-1, -2 under 500 IU/mL IFN-γ stimulatory conditions. Bar graphs show as the fold-change relative to sCD74 levels in supernatants of cells transfected with SC siRNA (n=3). (H) WB analysis of sCD74 in supernatants of A375, SK-MEL-2, and THP-1 MΦ with or without 100 nM BFA administration for 24 h under 500 IU/mL IFN-γ stimulation. (I) Release of sCD74 from A375, SK-MEL-2, and THP-1 MΦ was measured by ELISA after 24 h exposure to GW4869 (an exsome synthesis inhibitor) under 500 IU/mL IFN-γ stimulatory conditions. Bar graphs show as the fold-change relative to sCD74 levels in supernatants of cells treated with 0.5% DMSO (n=3). Graph values represent mean ± SD.

*ADAM* a disintegrin and metalloproteinase, *BFA* brefeldin A, *DMSO* dimethyl sulfoxide,

*ELISA* enzyme-linked immunosorbent assay, *IFN-γ* interferon-γ, *MΦ* macrophage, *SC* scramble, *SD* standard deviation,

*siRNA* short interference RNA, *WB* western blot, *3,4-DCI* 3,4-Dichloroisocoumarin

Supplementary Fig. 4


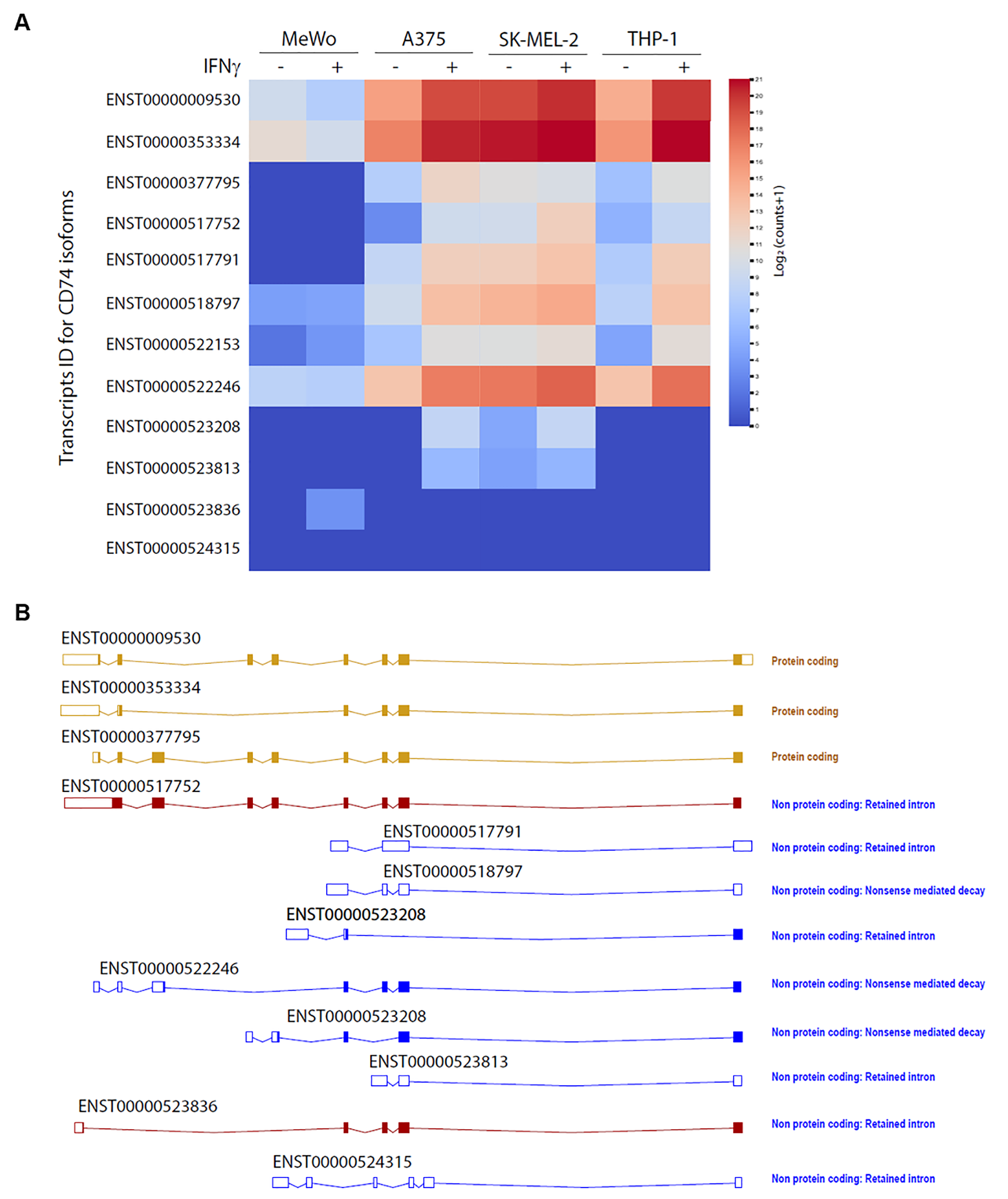


Supplementary Fig. 4 RNA sequence data for CD74 transcripts

(A) Heatmap of RNA sequence showing the identified transcripts for CD74 in MeWo, A375, SK-MEL-2, and THP-1 MՓ with or without 100 IU/mL IFN-γ stimulation. (B) Schematic diagrams showing the exon-intron structure of the identified transcript for CD74. Transcript IDs were acquired from Ensembl genome browser (<http://useast.ensembl.org/index.html>).

*IFN-γ* interferon-γ, *MΦ* macrophage

Supplementary Fig. 5


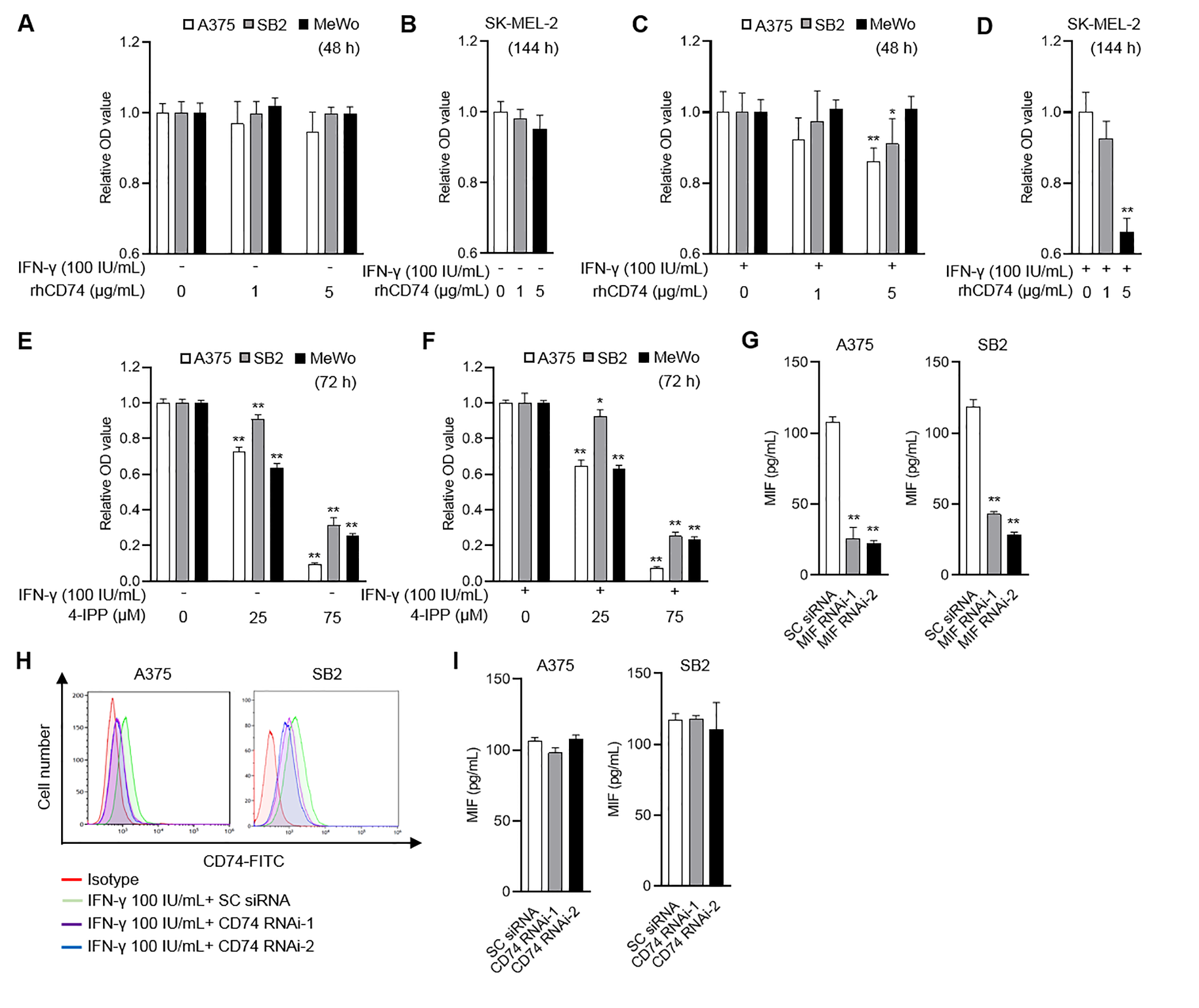


Supplementary Fig. 5 Impact of recombinant CD74 on melanoma cell growth

(A,B) Cell proliferation assay in A375, SB2, MeWo, and SK-MEL-2. Cells were treated with different concentrations of rhCD74 (0, 1, and 5 µg/mL) for 48 h in A375, SB2, and MeWo (A), or for 144 h in SK-MEL-2 (B) under basal conditions. Results represent the fold-change relative to the O.D. value of each cell line treated with 0 µg/mL rhCD74 (n=6). (C,D) Cell proliferation assay in A375, SB2, MeWo, and SK-MEL-2. Cells were treated with different concentrations of rhCD74 (0, 1, and 5 µg/mL) for 48 h in A375, SB2, and MeWo (C), or for 144 h in SK-MEL-2 (D) under 100 IU/mL IFN-γ stimulatory conditions. Results represent the fold-change relative to the O.D. value of each cell line treated with 0 µg/mL rhCD74 (n=6). (E,F) Cell proliferation assay in A375, SB2, and MeWo. Cells were treated with different concentrations of 4-IPP (0, 25, and 75 µM) for 72 h under basal conditions (E) or under 100 IU/mL IFN-γ stimulatory conditions (F). Results represent the fold-change relative to the O.D. value of each cell line treated with 0 µM 4-IPP (n=6). (G) Release of MIF in supernatants of A375, SB2 transfected with SC siRNA or MIF RNAi-1 or -2 measured by ELISA (n=4). (H) Flow cytometry assay for cell-surface CD74 in A375 and SB2 transfected with SC siRNA or CD74 RNAi-1 or -2 under 100 IU/mL IFN-γ stimulatory conditions. Histogram: red, isotype control; green, SC siRNA transfected cells stained with FITC-CD74 Ab; purple, CD74 RNAi-1 transfected cells stained with FITC-CD74 Ab; blue, CD74 RNAi-2 transfected cells stained with FITC-CD74 Ab. (I) Release of MIF in supernatants of A375, SB2 transfected with SC siRNA or CD74 RNAi-1 or -2 measured by ELISA (n=4). Graph values represent mean ± SD. Significance in difference between two groups was tested by Student *t*-test. **p* < 0.05, ***p* < 0.01.

*Ab* antibody, *DMSO* dimethyl sulfoxide, *ELISA* enzyme-linked immunosorbent assay, *IFN-γ* interferon-γ, *MIF* macrophage migration inhibitory factor, *MΦ* macrophage, *rh* recombinant human, *SC* scramble, *SD* standard deviation, *siRNA* short interference RNA, *4-1PP* 4-iodo-6-phenylpyrimidine

Supplementary Fig. 6

**
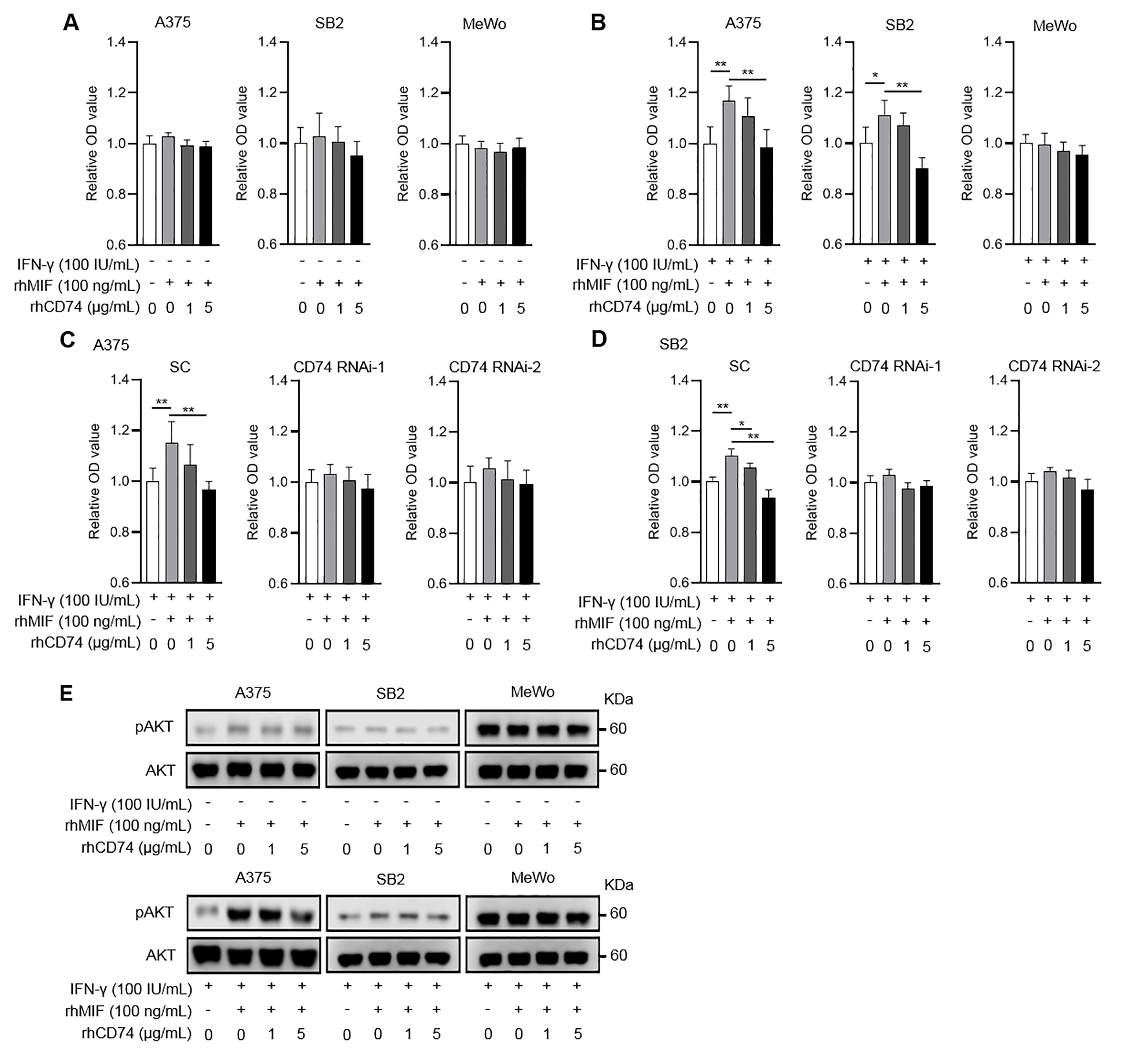
**

Supplementary Fig. 6 Impact of recombinant MIF and CD74 on melanoma cell growth

(A,B) Cell proliferation assay in A375, SB2, and MeWo. Cells were treated with rhMIF (0 and 100 ng/mL) and rhCD74 (0, 1, and 5 µg/mL) for 72 h under basal conditions (A) or under 100 IU/mL IFN-γ stimulatory conditions (B). Results represent the fold-change relative to the O.D. value of each cell line treated with 0 ng/mL rhMIF and 0 µg/mL rhCD74 (n=6). (C,D) Cell proliferation assay in A375 (C) and SB2 (D) transfected with SC siRNA and CD74 RNAi-1 and -2. Cells were treated with rhMIF (0 and 100 ng/mL) and rhCD74 (0, 1, and 5 µg/mL) for 72 h under 100 IU/mL IFN-γ stimulatory conditions. Results represent the fold-change relative to the O.D. value of each transfected cell treated with 0 ng/mL rhMIF and 0 µg/mL rhCD74 (n=6). (E) WB analysis of pAKT in A375, SB2, and MeWo 30min after rhMIF (0 and 100 ng/mL) and rhCD74 (0, 1, and 5 µg/mL) administration without IFN-γ stimulation (upper) or with 100 IU/mL IFN-γ stimulation (lower). AKT was used as a loading control. Graph values represent mean ± SD. Significance in difference between two groups was tested by Student *t*-test. **p* < 0.05, ***p* < 0.01.

*IFN-γ* interferon-γ, *MIF* macrophage migration inhibitory factor, *rh* recombinant human, *SC* scramble, *SD* standard deviation, *siRNA* short interference RNA, *WB* western blot

Supplementary Fig. 7

**
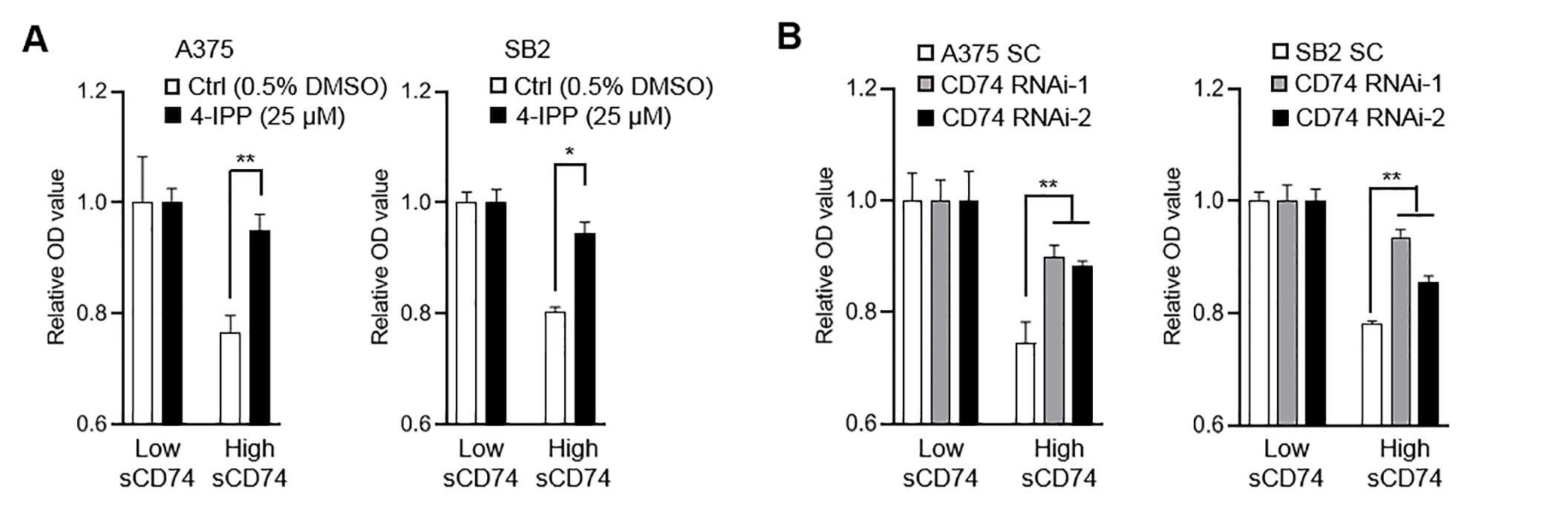
**

Supplementary Fig. 7 Impact of endogenous CD74 released by THP-1 MΦ on tumor growth

(A) Cell proliferation assay in A375 and SB2 48 h after co-culture with THP-1 MΦ and administration of 0 or 25 µM 4-IPP. Results represent the fold-change relative to the O.D. value of each cell line cultured in low sCD74-containing medium (n=4). (I) Cell proliferation assay in A375 and SB2 transfected with SC siRNA or CD74 RNAi-1 or -2. Cells were co-cultured with THP-1 MΦ for 48 h under 100 IU/mL IFN-γ stimulatory conditions. Results represent the fold-change relative to the O.D. value of each cell line cultured in low sCD74-containing medium (n=4). Graph values represent mean ± SD. Significance in difference between two groups was tested by Student *t*-test. **p* < 0.05, ***p* < 0.01.

*IFN-γ* interferon-γ, *MΦ* macrophage, *SC* scramble, *SD* standard deviation, *siRNA* short interference RNA, *4-1PP* 4-iodo-6-phenylpyrimidine

Supplementary Fig. 8

**
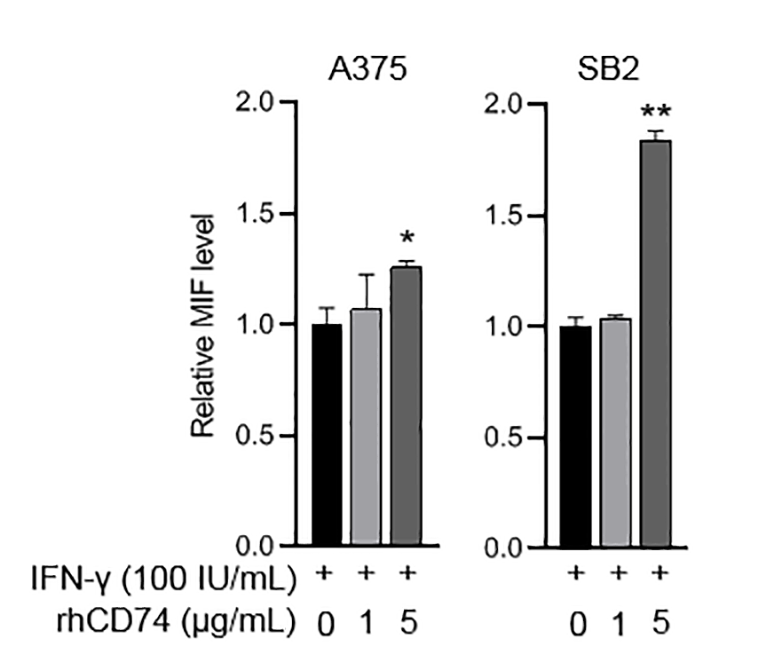
**

Supplementary Fig. 8 Release of MIF after rhCD74 administration

Release of MIF in supernatants of A375 and SB2 48h after treatment with different concentrations of rhCD74 (0, 1, and 5 µg/mL) under 100 IU/mL IFN-γ stimulatory conditions measured by ELISA (n=4), and the fold-change relative to MIF levels in supernatants of each cell line treated with 0 µg/mL rhCD74 is shown as bar graphs. Graph values represent mean ± SD. Significance in difference between two groups was tested by Student *t*-test. **p* < 0.05, ***p* < 0.01.

*ELISA* enzyme-linked immunosorbent assay, *IFN-γ* interferon-γ, *MIF* macrophage migration inhibitory factor, *MΦ* macrophage, *rh* recombinant human, *SD* standard deviation
