# Supplementary material for: Interplay between soluble CD74 and macrophage-migration inhibitory factor drives tumor growth and influences patient survival in melanoma
Source: Cell Death Dis. 2022 Feb 4;13(2):117. doi: 10.1038/s41419-022-04552-y (PMC8816905; doi:10.1038/s41419-022-04552-y)
Supplement: Supplementary file 3 — Supplementary Tables [file 41419_2022_4552_MOESM3_ESM.docx]

**SUPPLEMENTARY TABLES**

Table S1. Baseline characteristics of melanoma patients and normal healthy donors

| Characteristic | | | | Cohort 1  n=47 | Cohort 2  n=23 | Healthy donor  n=34 | |
| --- | --- | --- | --- | --- | --- | --- | --- |
| Age, years | | | | 62 (20-83) | 54 (32-74) | 58 (24-77) | |
| Gender, n (%) | | | |  |  |  | |
|  | | | Male | 27 (57.4) | 17 (73.9) | 24 (70.6) | |
|  | | | Female | 20 (42.6) | 6 (26.1) | 10 (29.4) | |
| Breslow thickness, mm | | | | 0.9 (0.2-12.0) | 3.0 (0.3-18.0) | - | |
| Ulceration, n (%) | | | |  |  |  | |
|  | | | Presence | 5 (10.6) | 13 (56.5) | - | |
|  | | | Absence | 33 (70.2) | 4 (17.4) | - | |
|  | | | Unknown | 9 (19.1) | 6 (26.1) | - | |
| LN metastases, n (%) | | | |  |  |  | |
|  | | Presence | | 9 (19.1) | 0 (0.0) | - | |
|  | | Absence | | 38 (80.9) | 23 (100.0) | - | |
| Pathological stage, n (%) | | | |  |  |  | |
|  | 0 | | | 6 (12.8) | 0 (0.0) | - |  |
|  | I | | | 23 (48.9) | 0 (0.0) | - |  |
|  | II | | | 9 (19.1) | 0 (0.0) | - |  |
|  | III | | | 9 (19.1) | 23 (100.0) | - |  |

Values are the number of patients (%) or the median (range), as indicated.

*LN* lymph node

Table S2. Properties of inhibitors

| Inhibitor | Target | Concentration |
| --- | --- | --- |
| GM6001 | Broad MMP and ADAM | 25 μM |
| GM1489 | Broad MMP | 25 μM |
| E-64 | Broad cysteine | 10 μM |
| Leupeptin | Broad serine, cysteine, and threonine | 50 μM |
| 3,4-DCI | Broad serine | 5 μM |
| β-secretase inhibitor IV | BACE1 and BACE2 | 10 μM |
| GI254023X | ADAM10 | 50 μM |
| TAPI-1 | ADAM17 | 50 μM |
| LY3000328 | Cathepsin S | 50 μM |
| Brefeldin A | Transport from ER to Golgi complex | 100 nM |
| GW4869 | Exosome synthesis | 10 μM |

*ADAM* a disintegrin and metalloproteinase, *ER* endoplasmic reticulum,

*MMP* matrix metalloproteinase, *3,4-DCI* 3,4-dichloroisocoumarin

Table S3. Antibodies used for Western blot

| Primary Ab  (clone no. for mAb) | Vendor | Catalog no. | Dilution |
| --- | --- | --- | --- |
| Actin (I-19) | Santa Crus Biotechnology | SC-1616 | 1:3000 |
| ADAM10 | Cell Signaling Technology | 14194S | 1:1000 |
| ADAM17 | Cell Signaling Technology | 3976S | 1:1000 |
| BCL-2 (50E3) | Cell Signaling Technology | 2870S | 1:1000 |
| Caspase-9 | Cell Signaling Technology | 9502 | 1:1000 |
| CD44 | Abcam | Ab157107 | 1:2000 |
| CD74* | R&D Systems | AF3590 | 1:200 |
| CD74 (PIN.1)** | Novus Biologicals | NB100-1985 | 1:1000 |
| CXCR2 | Proteintech | 20634-1-AP | 1:1000 |
| CXCR4 (12G5) | Invitrogen | 35-8800 | 1:250 |
| CXCR7 | Proteintech | 60216-1-Ig | 1:2000 |
| MIF | Proteintech | 20415-1-AP | 1:200 |
| Phospho-AKT Ser 473 | Cell Signaling Technology | 4060 | 1:1000 |
| AKT | Cell Signaling Technology | 9272 | 1:1000 |
| Phospho-BAD Ser 112 | Cell Signaling Technology | 5284 | 1:1000 |
| BAD | Cell Signaling Technology | 9292 | 1:1000 |
| Phospho-ERK1/2 Thr 202, Tyr 204 | Cell Signaling Technology | 9101 | 1:1000 |
| ERK1/2 | Cell Signaling Technology | 9102 | 1:1000 |

* The epitopes are present in regions of the ectodomain part of CD74.

** The epitope is present in a region of the cytoplasmic part of CD74.

Table S4. Target sequences for siRNA

| RNA name | Target sequence |
| --- | --- |
| ADAM10 RNAi-1 | CAUCUGACCCUAAACCAAA |
| ADAM10 RNAi-2 | CAAGGGAAGGAAUAUGUAA |
| ADAM17 RNAi-1 | GAAGAACACGUGUAAAUUA |
| ADAM17 RNAi-2 | UAUGGGAACUCUUGGAUUA |
| CD74 RNAi-1 | GACCUUAUCUCCAACAAUG |
| CD74 RNAi-2 | GGAGCUGUCGGGAAGAUCA |
| MIF RNAi-1 | GGGUCUACAUCAACUAUUA |
| MIF RNAi-2 | GCGCAGAACCGCUCCUACA |

*ADAM* a disintegrin and metalloproteinase, *MIF* macrophage migration inhibitory factor
